# Exploration of the biological mechanisms of CENPA as an oncogene in glioma: Screening based on cancer functional status

**DOI:** 10.1111/jcmm.70181

**Published:** 2024-12-02

**Authors:** Yuanguo Ling, Wei Teng, Niya Long, Wenjin Qiu, Ruting Wei, Yunan Hou, Lishi Jiang, Jian Liu, Xingwang Zhou, Liangzhao Chu

**Affiliations:** ^1^ Department of Neurosurgery The Affiliated Hospital of Guizhou Medical University Guiyang Guizhou China; ^2^ Department of Clinical Medicine Guizhou Medical University Guiyang Guizhou China; ^3^ Department of Neurosurgery Guizhou Provincial People's Hospital Guiyang Guizhou China

**Keywords:** cancer functional states, experiments in vitro, glioma, machine learning

## Abstract

Glioma is the most common primary tumour in central nervous system, characterized by high invasiveness, a high recurrence rate and extremely poor prognosis. Machine learning based on cancer functional state helps to combine multi‐omics methods to screen for key gene, such as CENPA, that influences the phenotype of glioma and patients' prognosis. Based on 14 CFS, glioma was divided into three subtypes. Bioinformatics and machine learning methods were utilized to develop an enhanced prognostic prediction signature based on three subtypes. We selected CENPA as a hub biomarker and conducted in vitro experiments such as IHC, western blot, Coip, transwell, cck8, flow cytometry, scratch assay, qPCR, AlphaFold, MOE and in vivo experiments. We identified three subtypes of glioma based on the 14 CFS. The C subtype exhibits poor clinical outcomes, increased carbohydrate and nucleotide metabolism, high infiltration of immune cells, high CNV and tumour mutation burden (*p* < 0.05). The differential expression of gene between three subtypes were used to construct a novel signature with improved performance in prognostic prediction via machine learning. CENPA was selected as the hub gene, in vitro experiments such as ihc, western blot and qPCR showed that CENPA had high expression in tissues and cell lines (*p* < 0.05). The scratch assay, edu, cck8, flow cytometry and transwell after CENPA knockdown or overexpression had significant effects on the functions of glioma. Meanwhile, CENPA was regulated by EZH2 and influenced downstream wnt pathway, affecting phosphorylation of two sites, Ser675 and Ser552, on β‐catenin. The effect of CENPA knockdown was reversed by drug CHIR‐99021. Animal experiments indicated that the tumour volume of control and overexpression group increased faster, especially the overexpression group, which was significantly faster (*p* < 0.001). Machine learning based on CFS is beneficial for the selection of key genes and disease assessment. In glioma, CENPA is positively correlated with WHO grading at both the gene and protein levels, and high CENPA affects patients' poor prognosis. Regulating CENPA can affect functions of glioma, and these phenomena may act through the EZH2/CENPA/β‐catenin signalling axis. CENPA knockdown can be reversed by the drug CHIR‐99021. CENPA may become one of the therapeutic targets in glioma.

## 1 INTRODUCTION

Glioma is a type of malignant brain  tumour that is commonly found in the brain and spinal cord and is one of the most aggressive forms of cancer in central nervous system.^1^, ^2^ Glioma, like other tumours, is characterized with heterogeneity.^3^ Glioma is further divided into molecular subtypes according to various factors, such as histological features and molecular signatures.^2^ These molecular subtypes are known to have distinct molecular and clinical characteristics, which have an impact on the prognosis and treatment in glioma. A better understanding of glioma will enable us to provide more accurate treatment for patients with glioma.

So far, more and more studies performed to explore the classification of glioma, but most of them were only focused on certain types of glioma features, such as tumour stem characteristic, inflammation, angiogenesis or epithelial‐mesenchymal transformation (EMT).^4^ In this study, a total of 14 malignant characteristics through CancerSEA (https://biocc.hrbmu.edu.cn/CancerSEA) were employed to perform unsupervised clustering analysis of gliomas.^5^ Glioma was classified into three subtypes based on cancer functional states. Each subtype had different clinical features, prognosis, immune infiltration, therapeutic response and somatic mutation. The differential expression of gene between those subtypes were used to construct a novel signature in prognostic prediction via machine learning.^6^ We further choose CENPA as hub gene to validate the models' precision and its important function.

CENPA, was the first discovered variant of histone H3, as a crucial component of centromeres, which are essential for proper chromosome segregation.^7^, ^8^, ^9^ Abnormal overexpression of CENPA was closely associated with the occurrence and development of multiple cancers.^10^, ^11^, ^12^, ^13^ This abnormal expression may be closely related to chromosomal instability, mitotic errors, cell cycle regulation and ultimately, tumour formation.^7^ However, research on CENPA in malignant tumours such as glioma was relatively limited. Given the significant role of CENPA in cancer biology, we aim to explore the function of CENPA, to better understand its potential role in glioma. At the same time, we still aim to clarify the significance of integrating cancer functional states with machine learning, providing new insights and strategies for glioma.

## 2 MATERIALS AND METHODS

### 2.1 Data preparation

In this study, the gene expression profile and corresponding clinical information of TCGA cohort were obtained from the UCSC website (http://xena.ucsc.edu/)differentially expressed Chinese Glioma Genome Atlas (CGGA) cohorts including mRNAseq_693, mRNAseq_325 and mRNA‐array_301 and corresponding clinical information were downloaded from CGGA website (http://www.cgga.org.cn/). We merged mRNAseq_693 and mRNAseq_325, and then named it CGGA1. The mRNA‐array_301 was defined as CGGA2. Furthermore, copy number variations and somatic mutation in TCGA‐LGGGBM cohort were downloaded from cbioportal website (https://www.cbioportal.org/). The methylation expression profile of TCGA cohort was obtained from UCSC website (http://xena.ucsc.edu/). The gene expression of normal brain tissues was extracted from the GTEX dataset via the UCSC website (http://xena.ucsc.edu/). We extracted 14 gene sets of cancer functional states from an online analysis website (CancerSEA; https://biocc.hrbmu.edu.cn/CancerSEA), including stemness, quiescence, angiogenesis, apoptosis, proliferation, cell cycle, metastasis, differentiation, DNA repair, hypoxia, EMT, inflammation, invasion and DNA damage.

### 2.2 GO and KEGG enrichments

Gene Ontology (GO) was utilized to annotate these genes, focusing on molecular functions (MF), biological processes (BP) and cellular components (CC). In addition, KEGG pathway enrichment analysis offered insights into high‐level genome functions. Both GO and KEGG analyses were performed using the ClusterProfiler package in software R, providing a deeper understanding of the oncogenic roles of the target genes.

### 2.3 Scoring assessment of gene sets

GSVA method in the GSVA package was used to qualify the 14 gene sets of cancer functional states.

### 2.4 Identification of subtypes based on cancer functional states (CFS)

We used 14 gene sets of cancer functional states to perform consensus clustering via the R package ‘ConsensusClusterplus’. TCGA‐LGGGBM cohort was classified into three subtypes according to the consensus heatmap and cumulative distribution function. CGGA1 and CGGA2 cohorts were used to validate consensus cluster based on the optimal k obtained from TCGA dataset.

### 2.5 Principal components analysis(PCA)

We used R software package (4.4.0). First, we performed Z‐score on the expression profile and further used prcomp function for dimensionality reduction analysis to obtain the matrix after dimensionality reduction.

### 2.6 Estimation of immune cell infiltration

The infiltration of immune cells was estimated by single‐sample gene‐set enrichment analysis (ssGSEA), MCPcounter and TIMER through R package ‘GSVA’, R package ‘MCPcounter’ and TIMER2.0 website, respectively.

### 2.7 Immune checkpoint blockade (ICB) therapy response

TIDE online algorithm (http://tide.dfci.harvard.edu/) was used to predict the ICB therapy response of TCGA‐LGGGBM cohort and then the therapy response of each CFS subtype were assessed according to the distribution of T‐cell dysfunction, exclusion and TIDE among CFS subtypes.

### 2.8 Gene set enrichment analysis (GSEA)

We calculated the fold changes of one subtype versus all other subtypes in the combined training dataset using the limma package before using GSEA.

### 2.9 Somatic mutation and copy number alteration analysis

The assessment of the CNV was carried out using the GISTIC algorithm. Copy number loss was defined as −1 and −2, while copy number amplifications were defined as 1 and 2. The absence of a CNV event was defined as 0. The clinical information provided on the Cbioportal website was the direct source for extracting the information related to the 1p19q codeletion. The utilization of the onco‐print tool from the ‘complexheatmap package’ allowed for the visualization of somatic mutation and copy number alteration within CFS subtypes. The basis for computing tumour mutation burden (TMB) is the quantity of mutations that are not silent.

### 2.10 Construction and validation of the risk model via machine learning

First, we applied the R packages ‘Samr’ and ‘Veen’ to identify intersect genes with differentially expression among CFS subtypes from TCGA, CGGA1 and CGGA2 datasets. We integrated 10 machine learning algorithms with 101 algorithm combinations to achieve a highly accurate and stable performance for the CFS consensus development. The algorithms integrated into the analysis were RSF, Enet, Lasso, Ridge, stepwise Cox, CoxBoost, plsRcox, SuperPC, GBM and survival‐SVM. To generate the signature, prognostic intersection genes were identified using univariate Cox regression in the TCGA‐LGGGBM cohort. Then, 101 algorithm combinations were used to build prediction models based on leave‐one‐out cross‐validation (LOOCV) in the same cohort. These models were then validated in two other datasets (CGGA1 and CGGA2). The concordance index (C‐index) based on Harrell's method was calculated for each model across all validation datasets, and the model with the highest average C‐index was considered optimal.

### 2.11 IHC

4‐μm tissue sections were subjected to microwave heating in 10 mM sodium citrate solution for antigen retrieval and then quenched with 3% H_2_O_2_ for 10 min. After blocking with goat serum, the sections were incubated with primary antibodies overnight at 4°C, followed by incubation with secondary antibodies for 1 h at 37°C. The signals on the sections were visualized using a diaminobenzidine chromogen (Dako North America, Carpinteria, USA). Images were captured using a light microscope (Olympus, Tokyo, Japan) and analysed with ImageJ 1.8.0 software.

### 2.12 Cell culture

The LN229 and U251 cells were purchased from Procell (Wuhan, China). Glioma cells were cultured using Dulbecco's Modified Eagle's Medium (DMEM, USA), which contained 10% foetal bovine serum (FBS, Gibco, Australia) and 1% penicillin–streptomycin (Israel). The cells were grown at 37°C and 5% CO_2_.

### 2.13 Western blot

Cells were harvested and lysed using RIPA lysis buffer (Cell Signal Technology, Danvers, MA, USA). The extracted proteins were resolved using 10% SDS‐PAGE and transferred to a PVDF membrane (Millipore, Billerica, MA, USA). After blocking with 5% defatted milk for 1 h at room temperature, membranes were incubated overnight at 4°C with Anti‐CENPA Antibody (1:5000, solarbio). The next day, membranes were washed with TBST twice and incubated with HRP Conjugated Goat anti‐Rabbit IgG Polyclonal Antibody (1:5000 dilution, Huabio, China) for 1 h at room temperature. The signals were detected using ECL reagent (Millipore, Billerica, USA) and visualized using Image Lab software 5.2(Bio‐Rad, Hercules, USA).

### 2.14 Co‐IP

To evaluate protein interactions, a Co‐IP assay was performed according to beyotime manufacturer (Wuhan, China).

### 2.15 RNA extraction and qPCR

The RNA was extract according Tiangen's TRNzol Universal Reagent (Beijing, China). And reverse‐transcribed following the standard method (Takara, Kyoto, Japan). The primer information are listed in Table 1.

**TABLE 1:** The sequences of primers.

Primers	Leading strand	Lagging strand
CENPA	TCCATCATATAGACCTCTGCCCTTC	ACACATCCGTTGACAAGCACAG
GAPDH	GATTCCACCCATGGCAAATTC	CTGGAAGATGGTGATGGGATT
GPX8	GCCCAGAGCAAAGGTTTCACTA	TTTGTGCAGTTCCTTCAGCCC
COL3A1	TGCCCTACTGGTCCTCAGAA	AGATATCCCAGCTGGACCTT
GRP65	AATGTGACTTTTGAAATGCC	ATAGACTAAGAGGTGGAGGC
SRPX2	GAGCTTGACAAAGTGGTCGT	CCACATGCTACTCACCGAAGG
EZH2	AATCAGAGTACATGCGACTGAGA	GCTGTATCCTTCGCTGTTTCC

### 2.16 Cell Counting Kit‐8

Cck‐8 (Solarbio, China) was used following the instructions, and observations are made according to a time gradient. The results are measured using an Enzyme‐labelled instrument (Thermofisher, USA).

### 2.17 Transwell assay

After coating the chambers with or without 50 μL Matrigel (1:15 dilution) for 16 h at 37°C, the cells were cultured in upper transwell chambers (8 mm pore, Corning, USA). A total of 3 × 104 cells were seeded in the upper compartments for Cenpa silencing, while 2 × 104 cells were seeded for Cenpa overexpression. The upper compartments contained 400 μL DMEM, while the lower wells contained 1 mL DMEM with 10% FBS. After 36 h, the cells that migrated to or invaded the lower chambers were stained with 0.5% crystal violet and counted. Using ImageJ and GraphPad Prism 8.3 for data processing and statistical analysis.

### 2.18 Scratch assay

Cells were cultured in 6‐well plates until they reached 80–90% confluency. A spearhead was employed to create scratches on the cell monolayer, and photographs of the resulting ‘wound’ were captured. After 24 or 48 h, a fresh set of images were taken and compared to the previous ones.

### 2.19 Cell transfection

Transfection of LN229 and U251 was performed according to the Lip3000 (Invitrogen, USA). The qPCR and WB were to detect transfection efficiency. Sequence information for transcript NM_001042426.2 was obtained through the NCBI website. The CENPA sequence was designed using the shRNA design website as follows: ShRNA‐1: AGACAAGGTTGGCTAAACCTC, ShRNA‐2: GCAGCAGAAGCATTCTAGTT, ShRNA‐3: CCGAGTTACTCTCTTCCCAAA. For the overexpression of CENPA, oligo sequences were designed using a primer design website: h‐CENPA‐MluI‐1F: TCTCGAGAATTCTCACGCGTC CCTCTGCGGCGTGTCC. h‐CENPA‐NotI‐1R: AAGTTAGTAGCGGC GGCCGCGCCGAGTCCCTCCTCAAG. The EZH2 siRNA forward sequence is CCUGACCUCUGUCUUACUU, and the reverse sequence is AAGUAAGACAGAGGUCAGG.

### 2.20 Edu assay

The edu experiments were used according to BeyoClick™ EdU‐555 Cell proliferation test kit.

### 2.21 Flow cytometry

The rate of apoptosis and cell cycle in LN229 and U251 was determined using a Propidium iodide (PI)/annexin V‐FITC kit and cell cycle kit (Multisciences, China).

### 2.22 AlphaFold

The structure of EZH2 and CENPA proteins was predicted using the multimer model in Alphafold v2.3.2. Default parameters were used, and the database versions used were as follows: UniProt and UniRef90 from 01 March 2023, PDB_MMCIF and PDB_SEQRES from 03 March 2023, while the versions of other databases were default. Predicted multimer images were obtained using PyMOL v2.5.0. The predicted multimeric structure was analysed using PRODIGY v2.0 to determine the binding affinity and dissociation constant of the multimer. Note: A dissociation constant between the two proteins less than 10^−8^ indicates strong binding, less than 10^−4^ but greater than 10^−8^ indicates moderate binding, and greater than 10^−4^ indicates weak binding. Arpeggio was used to analyse the predicted multimeric structure and obtain information about interactions within the multimer. Regarding the calculation of the binding affinity, we have used the molar Gibbs free energy (ΔG, binding free energy) to determine the affinity (dissociation constant Kd or binding constant Ka).

### 2.23 Statistical analysis

All data processing, statistical analysis and plotting were conducted in R 4.4.0 and graphpad prism 8.0 software. Correlations between two continuous variables were assessed via Pearson's correlation coefficients. The chi‐squared test was applied to compare categorical variables, and continuous variables were compared through the Wilcoxon rank‐sum test or *T* test. The survminer package was used to determine the optimal cut‐off value. Cox regression and Kaplan–Meier analyses were performed via the survival package. The receiver operating characteristic curve (ROC) used to predict binary catego rical variables was implemented via the pROC package. The time‐dependent area under the ROC curve (AUC) for survival variables was conducted by the timeROC package. All statistical tests were two‐sided. *p* < 0.05 was regarded as statistically significant.

## 3 RESULTS

### 3.1 Identification of three subtypes based on CFS

**FIGURE 1:**
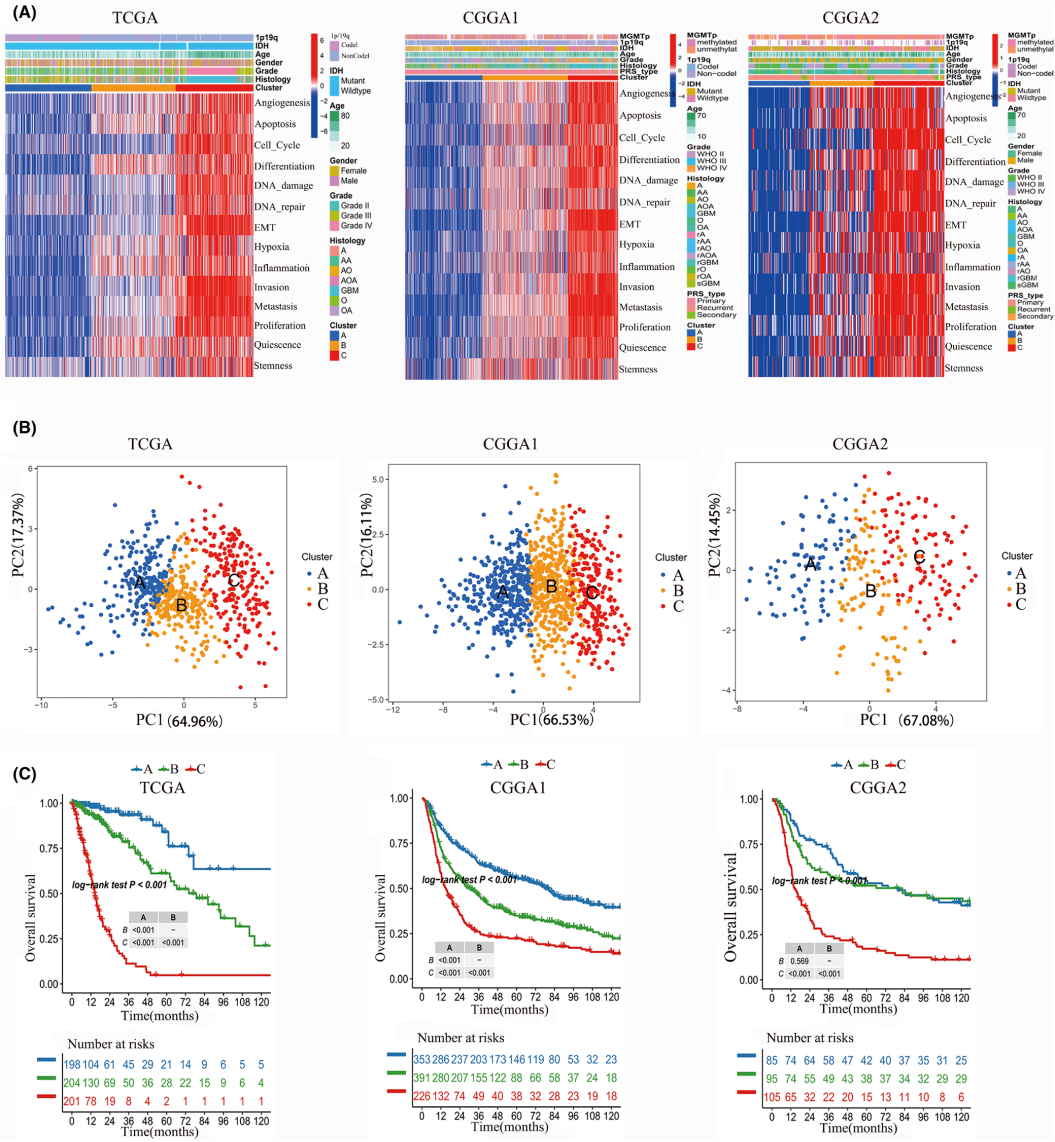
The subtype of glioma based on CFS. (A) The glioma of TCGA, CGGA1 and CGGA2 cohorts were divided into three glioma subtypes based on the cancer functional status scores calculated by the GSVA method via consensus clustering. (B) PCA analysis confirmed that the three subtypes of TCGA, CGGA1 and CGGA2 datasets can be distinguished significantly. (C) Survival analysis of three subtypes in TCGA, CGGA1 and CGGA2 cohorts between three subtypes, respectively.

We identified three subtypes of glioma (A, B and C) based the 14 CFS via consensus clustering method (Figure 1A). The results of principal component analysis (PCA) revealed that there was a significant separation between subtype A and subtype C, on the other hand, subtype B was observed to be distributed between the two subtypes, indicating that it may be classified as a category of transition features (Figure 1B). Survival analysis indicated that subtype A have the best prognosis, while subtype C may suffer from the shortest overall survival (OS). Regarding with subtype B, the curve shows that the OS is between A and C. We observed that those 14 CFS were high expression in subtype C, but low expression in subtype A. CSF expression in subtype B falls between that of subtypes A and C (Figure 1C). Furthermore, malignant characteristics such as high grade, GBM, IDH1 wildtype, 1p19q non‐codeletion and advanced age are enriched in subtype C. Subtype B is the opposite of subtype C (Figure 1A). We also validate this CFS subtypes in CGGA1 and CGGA2, respectively, and the similar results were observed (Figure 1B,C).

### 3.2 Immune infiltration and diverse somatic variations of CFS subtypes

**FIGURE 2:**
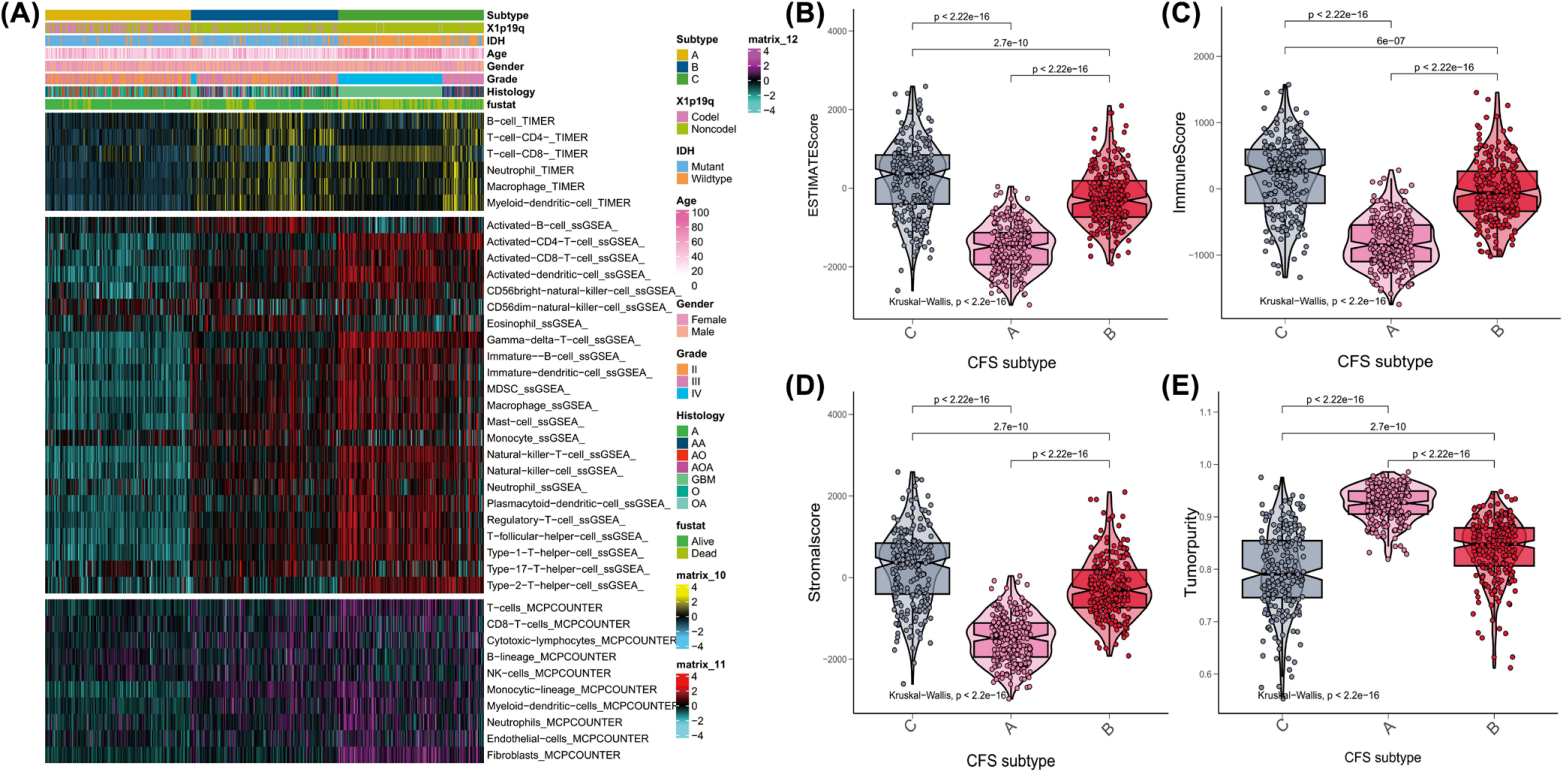
Immune infiltration of CFS subtypes. The heatmap showed that the immune infiltration of CFS subtypes calculated by ssGSEA, MCPcounter and TIMER (A). The estimatescore (B), immunescore (C), stromalscore (D) and tumorpurity (E) among CFS subtypes.

We found that subtype C was associated with immune‐related signature, such as T cell receptor signalling pathway, intestinal lgA production immune network. So, we used ssGSEA, MCPcounter and TIMER to assess the immune infiltration of CFS subtypes and found that subtype C had a high enrichment of immune cells (Figure 2A). The immune, stromal and ESTIMATE scores of subtype C exceeded those of subtypes A and B, but the tumour purity was lower in subtype C than in subtypes A and B (Figure 2B–D). Similarly, subtype B had higher immune, stromal and ESTIMATE scores compared to subtype A, but the tumour purity in subtype B was lower than that of subtype A (Figure 2E).

Furthermore, we also assessed the mRNA, mRNA vs. methylation, amplification frequency and deletion frequency of 75 immunomodulators among CFS subtypes, respectively (Figure S1A–E). This study analysed the possible somatic changes that may occur in CFS subtypes, and identified the most frequent genetic mutations or disruptions in critical pathways and CNAs (Figure S2A–F).

### 3.3 Construction and validation of the risk model via machine learning

**FIGURE 3:**
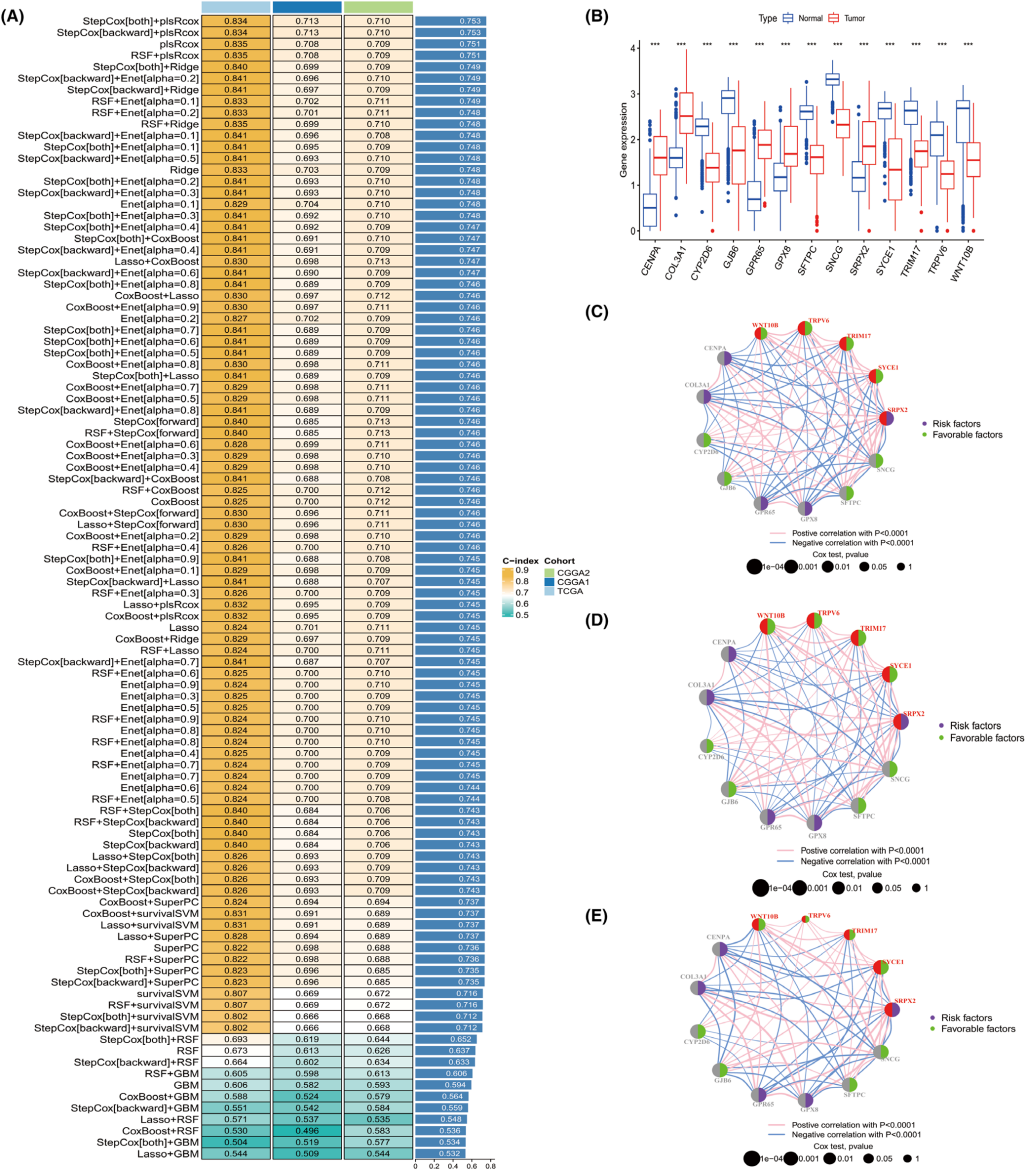
Thirteen genes were identified by machine learning‐based integrative procedure. The C‐index of each model across all validation datasets (A); The expression of those 13 genes between tumour and normal tissues (B); The prognostic role of those 13 genes in glioma of TCGA (C), CGGA (D) and CGGA2 (E) datasets, respectively.

We combined 10 machine‐learning algorithms into 101 combinations which has been proven effective in autonomously selecting pivotal genes from whole transcriptomic profiles to develop dependable cancer prognostic models.^14^ The 10 algorithms integrated into the analysis were RSF, Enet, Lasso, Ridge, stepwise Cox, CoxBoost, plsRcox, SuperPC, GBM and survival‐SVM. We applied the R packages ‘samr’ and ‘Veen’ among different CFS subtypes and took the insection genes obtained from the TCGA, CGGA1 and CGGA2 datasets. A total of 301 intersection genes were obtained. Our machine learning‐based integrative procedure was applied to these 301 intersection genes to generate a consensus CFS‐related signature. One hundred and one prediction models were fitted using the LOOCV framework on the TCGA dataset. The C‐index of each model was subsequently calculated across all validation datasets (Figure 3A). The combination model of StepCox [both] and plsRcox had the highest average C‐index of 0.753 and was found to be the optimal model (Figure 3A). Furthermore, this combination model showed the highest C‐index among all validation datasets. The combination model of StepCox [both] and plsRcox identified 13 genes, including CENPA, COL3A1, CYP2D6, GJB6, GRP65, GPX8, SFTPC, SNCG, SRPX2, SYCE1, TRIM17, TRPV6 and WNT10B. CENPA, COL3A1, GJB6, GRP65, GPX8, SFTPC, SNCG, SRPX2, SYCE1 and TRIM17. CENPA, COL3A1, GRP65, SRPX2 and GPX8 were upregulated in glioma, while remaining genes were downregulated (Figure 3B). All of those 13 genes were associated with prognosis of patients with glioma (Figure 3C,D).

The risk model was constructed by the combination of StepCox [both] and plsRcox based on the above 13 genes in TCGA dataset and validated in CGGA1 and CGGA2 cohorts, respectively. We revealed that CENPA, COL3A1, GRP65, SRPX2 and GPX8 were high expression in high riskscore group, while the remaining genes were low expression in low riskscore group (Figure S3A–C). Survival analysis indicated that patients with high riskscore experienced shorter OS and multivariate analysis suggested that riskscore was an independent factor for predicting prognosis (Figure S3D–F). We further observed that high riskscore was associated with malignant characteristics, such as high‐grade glioma, 1p19q non‐codeletion, IDH1 wildtype (Figure S4A–I, Figure S5A–C). A nomogram was used to visualize the final multivariable logistic regression model (Figure S6A,B). The riskscore exhibited remarkable predictability as compared to IDH, 1p19q, tumour grade and age. Moreover, the model relying on age, tumour grade, IDH mutation, 1p19q codeletion and riskscore demonstrated a higher concordance index compared to the remaining models (Figure S6C). Calibration curves were drawn and the TCGA cohort curve matched the expected outcome (Figure S6D–F). We also validated those results in CGGA1 and CGGA2 datasets and the similar results were obtained (Figure S7A–F).

### 3.4 Exploration of CENPA in glioma

**FIGURE 4:**
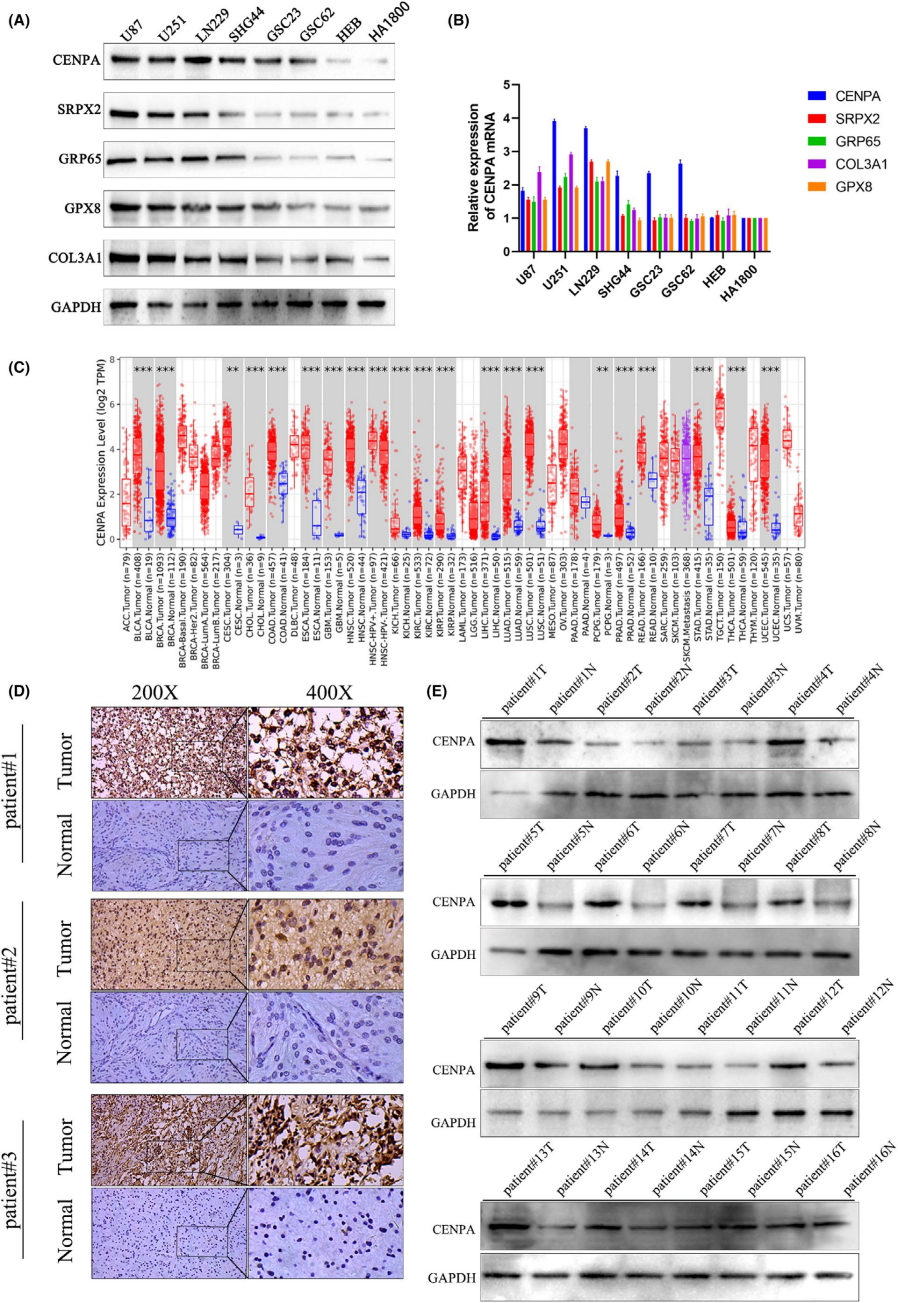
The expression of CENPA in glioma. (A) The protein expressions of five genes in glioblastoma cell lines and normal astrocyte lines. (B) The qPCR in glioblastoma cell lines and normal astrocyte lines. (C) Pan‐cancer analysis of CENPA in TCGA. (D) The expression of CENPA in glioma and normal tissues through IHC. (E) The western blot detected the CENPA protein in tissues.

Through western blot and qPCR on U87, U251, LN229, SHG44, GSC62, GSC23, HEB and HA1800 cell lines, we confirmed that in these cell lines, COL3A1, GRP65, SRPX2 and GPX8 showed different trends at the gene level between cancer and normal tissues (Figure 4A,B). The expression of these four genes at the protein level was also unstable, in contrast, CENPA showed a consistent upregulation trend at both the gene and protein levels (Figure 4A,B). Considering the important role of CENPA in centromere proteins and its current progress in glioma, we chose to further study CENPA. To verify this hypothesis, we focused on the expression and function of CENPA in further experiments.

**FIGURE 5:**
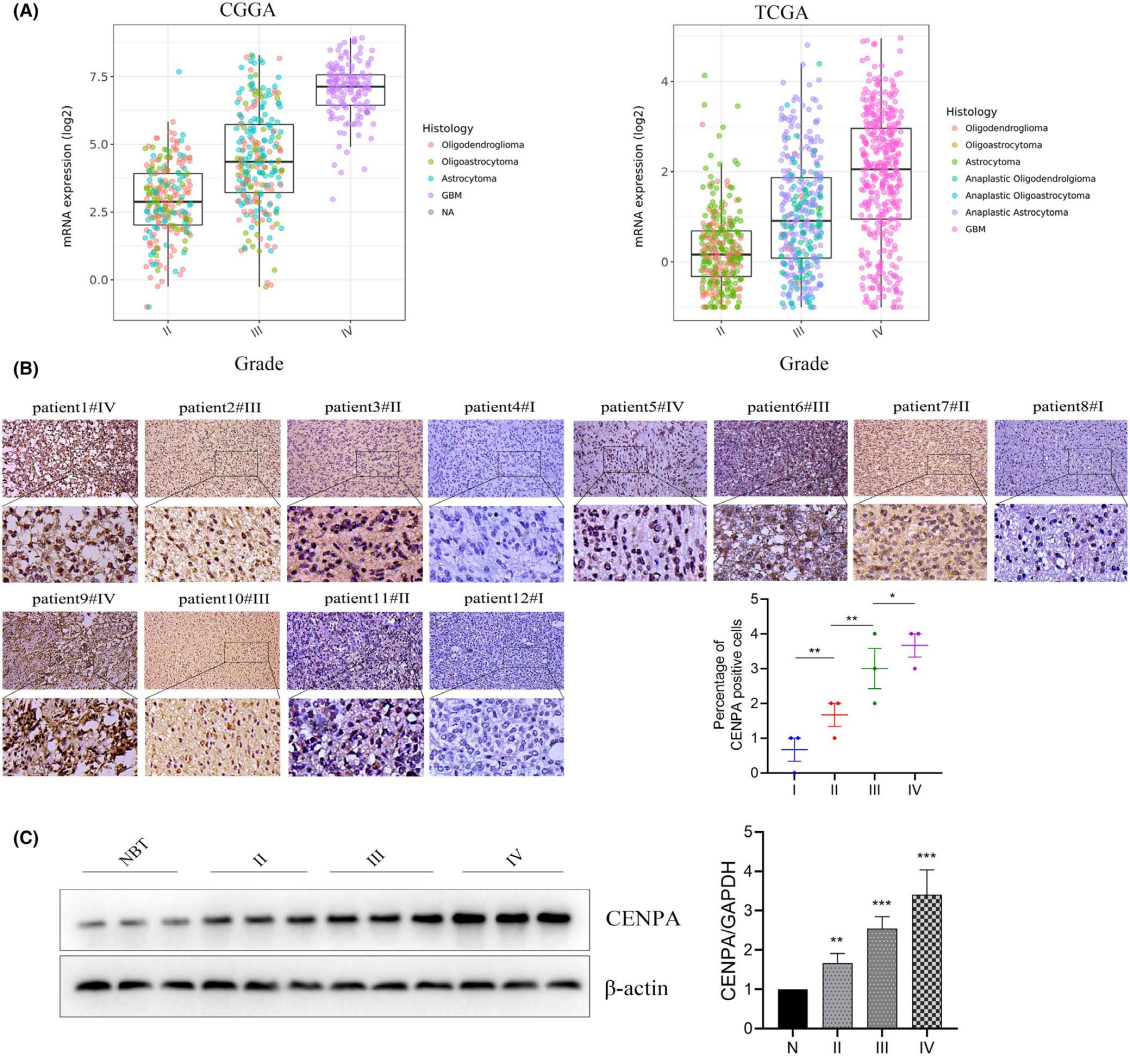
Expression of CENPA across different grades of glioma. (A) Expression profiles of CENPA in glioma of various grades from TCGA and CGGA datasets. (B) Immunohistochemistry was employed to examine samples of glioma from different grades. (C) Western blot analysis was conducted to assess the expression of CENPA in glioma of various grades.

Using sequencing data from various cancers in the TCGA database, we conducted a pan‐cancer analysis of CENPA. We found that CENPA was upregulated in multiple tumours, including glioma, bladder cancer, breast cancer and liver cancer, among others which indicated that CENPA was widely expressed in various cancers and showed mRNA upregulation in both high‐grade and low‐grade glioma (*p* < 0.001, Figure 4C). Furthermore, we conducted experiments using cancer and adjacent non‐cancerous tissues from 102 glioma patients. Through immunohistochemistry (IHC) and western blot experiments, we found that CENPA was upregulated in glioma, with a significant difference observed statistically (*p* < 0.001, Figure 4D,E). Analysis of data from TCGA and CGGA revealed a gradual upregulation of CENPA gene expression with increasing glioma grade (*p* < 0.001, Figure 5A). Moreover, by validating the immunohistochemistry results, we confirmed the same trend observed in the TCGA and CGGA databases (Figure 5B). Additionally, western blot analysis of CENPA protein levels showed a positive correlation with WHO grading, consistent with the results from bioinformatics analysis (*p* < 0.001, Figure 5C).

### 3.5 The regulation of CENPA affected the function of glioma cells

**FIGURE 6:**
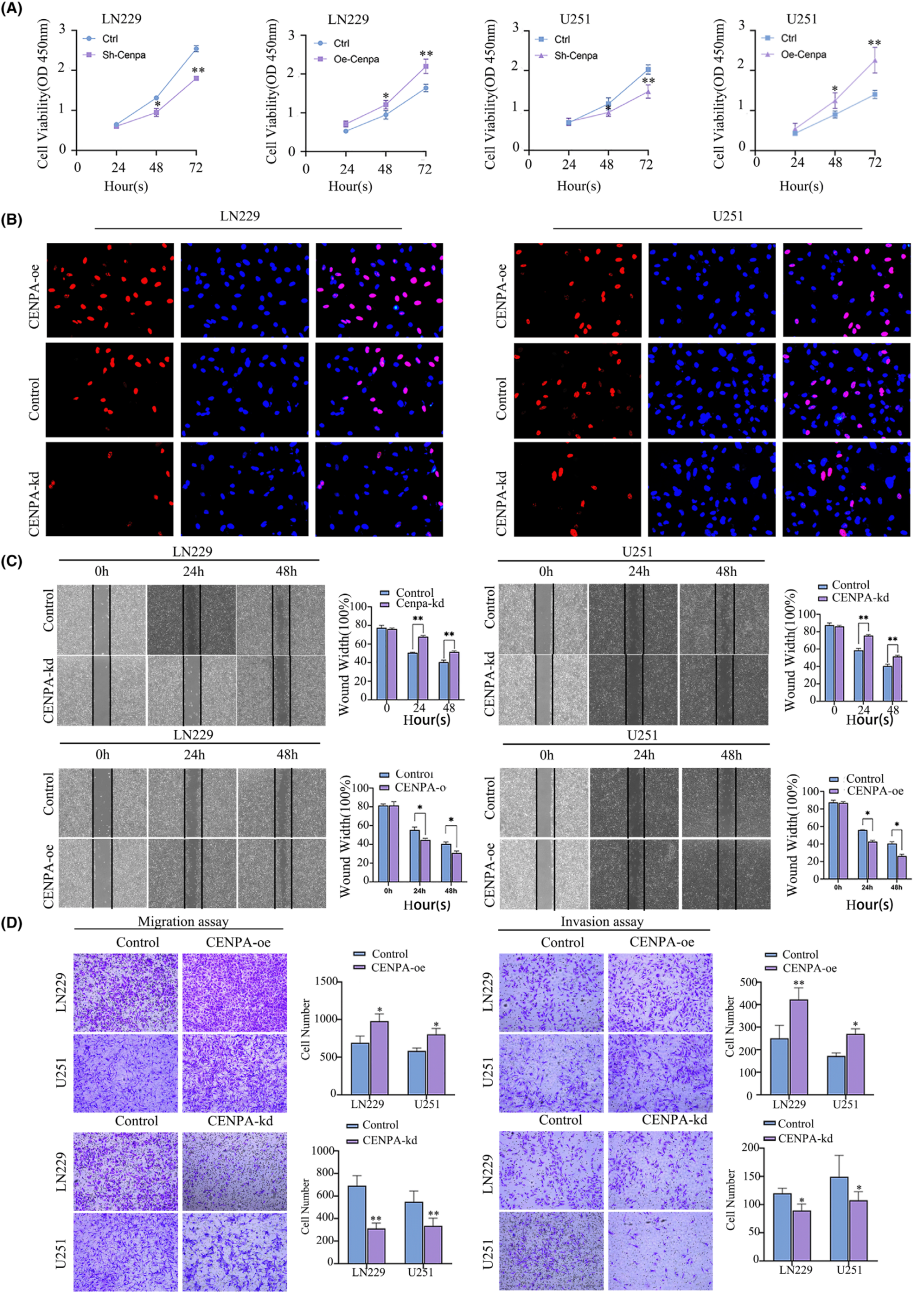
The regulation of CENPA influenced the proliferation, migration and invasion. (A) The CCK8 assay kit evaluated cell proliferation after transfection of two cell lines. (B) The EDU assay measured cell proliferation at the fluorescence level. (C) Scratch assay conducted post‐transfection. (D) Transwell assay was utilized to assess the invasive and migratory capacities of transfected U251 and LN229 cells.

U251 and LN229 cell lines were selected for transfection, and the transfection efficiency was detected by western blot and qPCR (*p* < 0.05, Figure S8). Based on the transfection efficiency, the CENPA#sh3 knockdown group was selected. The CCK8 results showed that within 48 and 72 h, a significant decrease in cell proliferation rate was observed after CENPA knockdown, while a significant increase in proliferation rate was observed at 24 h in LN229 and U251 cells overexpressing CENPA, which remained significant at 48 and 72 h (*p* < 0.001, Figure 6A). In the CENPA knockdown cell lines, the EDU assay showed a significant decrease in cell red fluorescence compared to the control group after 48 h of CENPA knockdown (*p* < 0.001, Figure 6B).

The migration and invasion of glioma cells reflected their malignancy. Scratch assays indicated that within 48 h post‐transfection, U251 and LN229 cell lines were significantly affected. When CENPA was knocked down, both cell lines showed similar results: the migration rate of the cell lines decreased significantly within 48 h. Conversely, when CENPA was overexpressed, the migration rate of the cell lines increased (*p* < 0.001, Figure 6C). Additionally, Transwell assays also showed a significant decrease in the number of cells passing through to the lower layer when CENPA expression was reduced, while overexpression of CENPA enhanced migration ability. Furthermore, when Matrigel was added, the invasion ability of CENPA‐knockdown cell lines decreased, indicating a possible impact of CENPA knockdown on matrix metalloproteinases (*p* < 0.001, Figure 6D).

**FIGURE 7:**
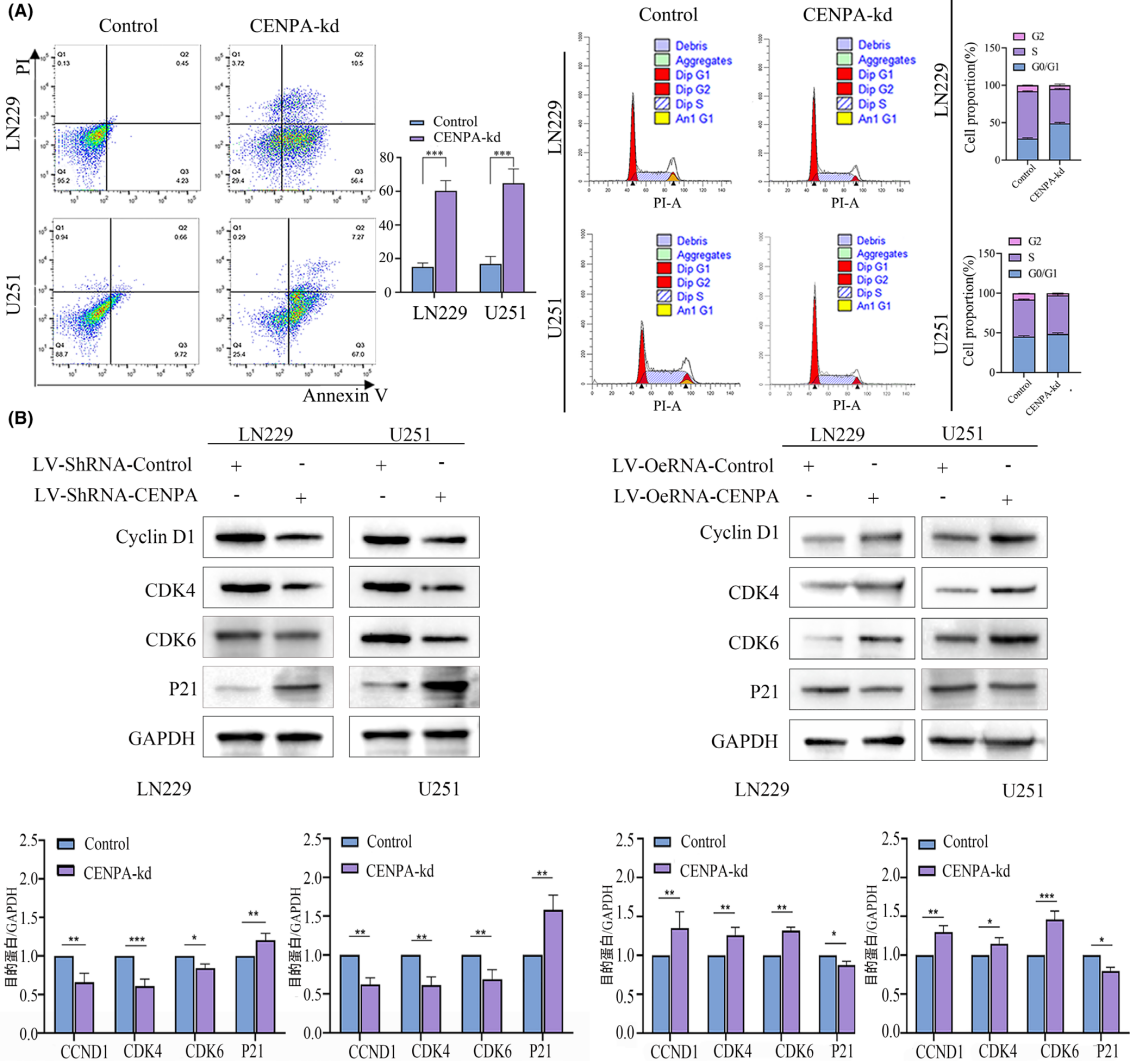
CENPA affected the cell cycle and proapoptosis in glioma. (A) CENPA knockdown led to cell cycle arrest and proapoptosis. (B) CENPA‐influenced cell cycle‐related proteins.

After knocking down CENPA, we observed significant effects in the LN229 and U251 cell lines. The proportion of tumour cells entering the G2 and S phases decreased in the knockdown group, with most cells arrested in the G1 phase, indicating that CENPA might affect mitosis in the G1 phase (Figure 7A). Within 24 h of knocking down CENPA, glioma cells showed significant cell death, with both early and late apoptosis occurring, and a higher proportion of early apoptosis (*p* < 0.05, Figure 7A). Western blot results showed a significant decrease in the expression of CCND1, CDK6, CDK4 and an increase in the expression of the tumour suppressor protein p21 in LN229 and U251 cells after knocking down CENPA, suggesting that knocking down CENPA might induce cell apoptosis (*p* < 0.05, Figure 7B). Enrichment analysis indicated that CENPA could affect the p53 signalling pathway. Combined with the changes in p21, it was inferred that CENPA might affect the cell cycle and induce cell apoptosis through the p53‐p21 axis.

### 3.6 CENPA influenced the glioma through EZH2/CENPA/Wnt pathway

**FIGURE 8:**
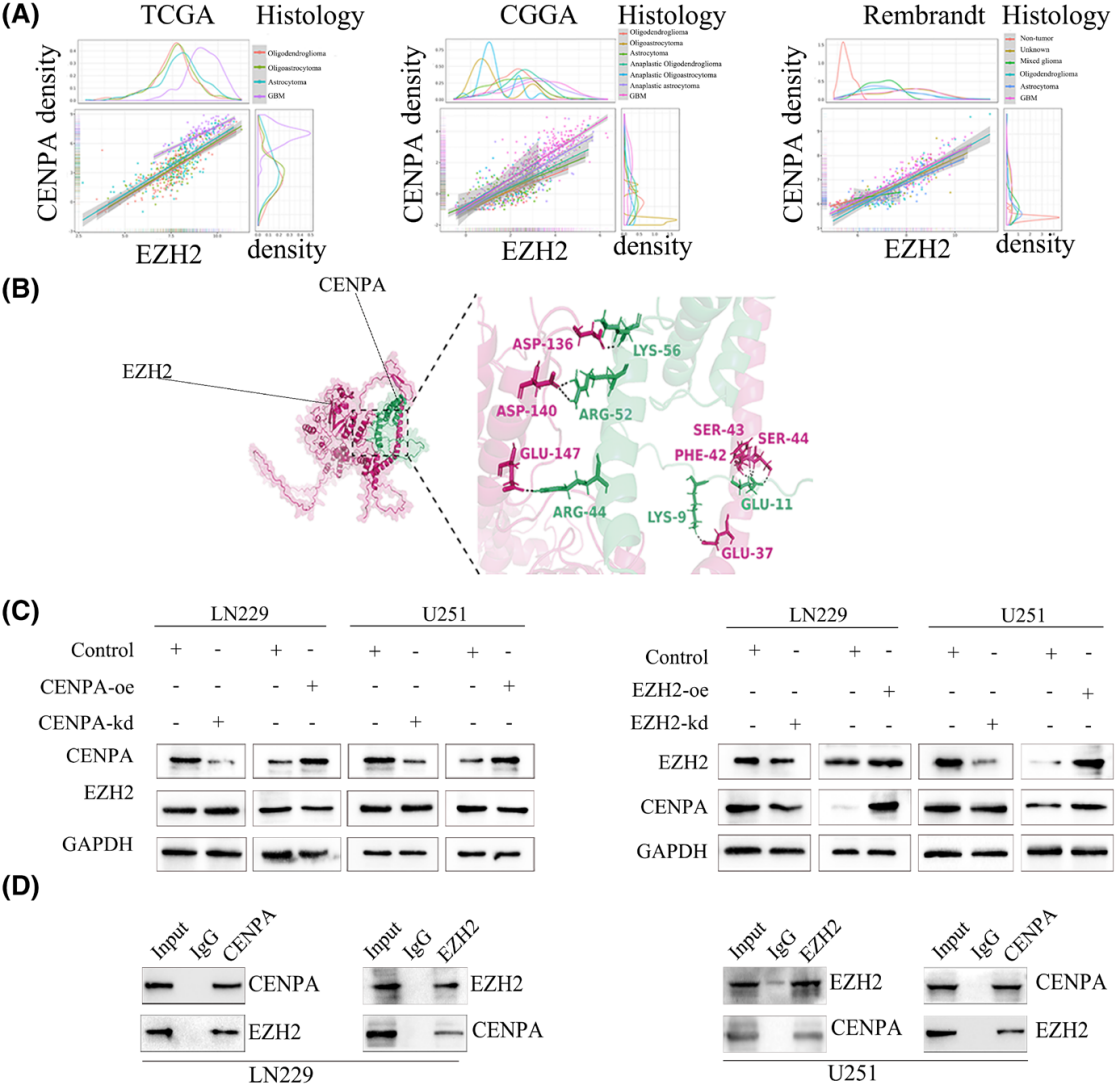
The correlation with EZH2 and CENPA. (A) The relationship with CENPA and EZH2 in TCGA, CGGA, Rembrandt datasets. (B) Using AlphaFold software v2.3.2 to explore the correlation with CENPA and EZH2. (C) The relationship between EZH2 and CENPA after transfection were detected by western blot. (D) Coip was further used to validate the relationship between CENPA and EZH2.

To further investigate the mechanism of CENPA in glioma, we searched for transcription factors that might bind to CENPA and found Enhancer of Zeste Homologue 2 (EZH2). Through analysis of TCGA, Rembrandt and CGGA databases, we discovered a close and statistically significant relationship between EZH2 and CENPA in glioma of all grades (Figure 8A). Using AlphaFold protein–protein docking software, we found that the binding affinity between EZH2 and CENPA was −10.7 kcal·mol^−1^, with a dissociation constant of 1.5e‐08, indicating a tight binding (Figure 8B). After transfection of CENPA and EZH2, we found that knocking down CENPA did not significantly affect the gene and protein levels of EZH2. However, when EZH2 was knocked down, the RNA and protein expression levels of CENPA showed a decreasing trend, while overexpression showed the opposite result (Figure 8C). Considering the transcriptional role of EZH2, it is likely an upstream regulatory factor of CENPA. Immunoprecipitation experiments further confirmed the interaction between EZH2 and CENPA in U251 and LN229 cells (Figure 8D).

**FIGURE 9:**
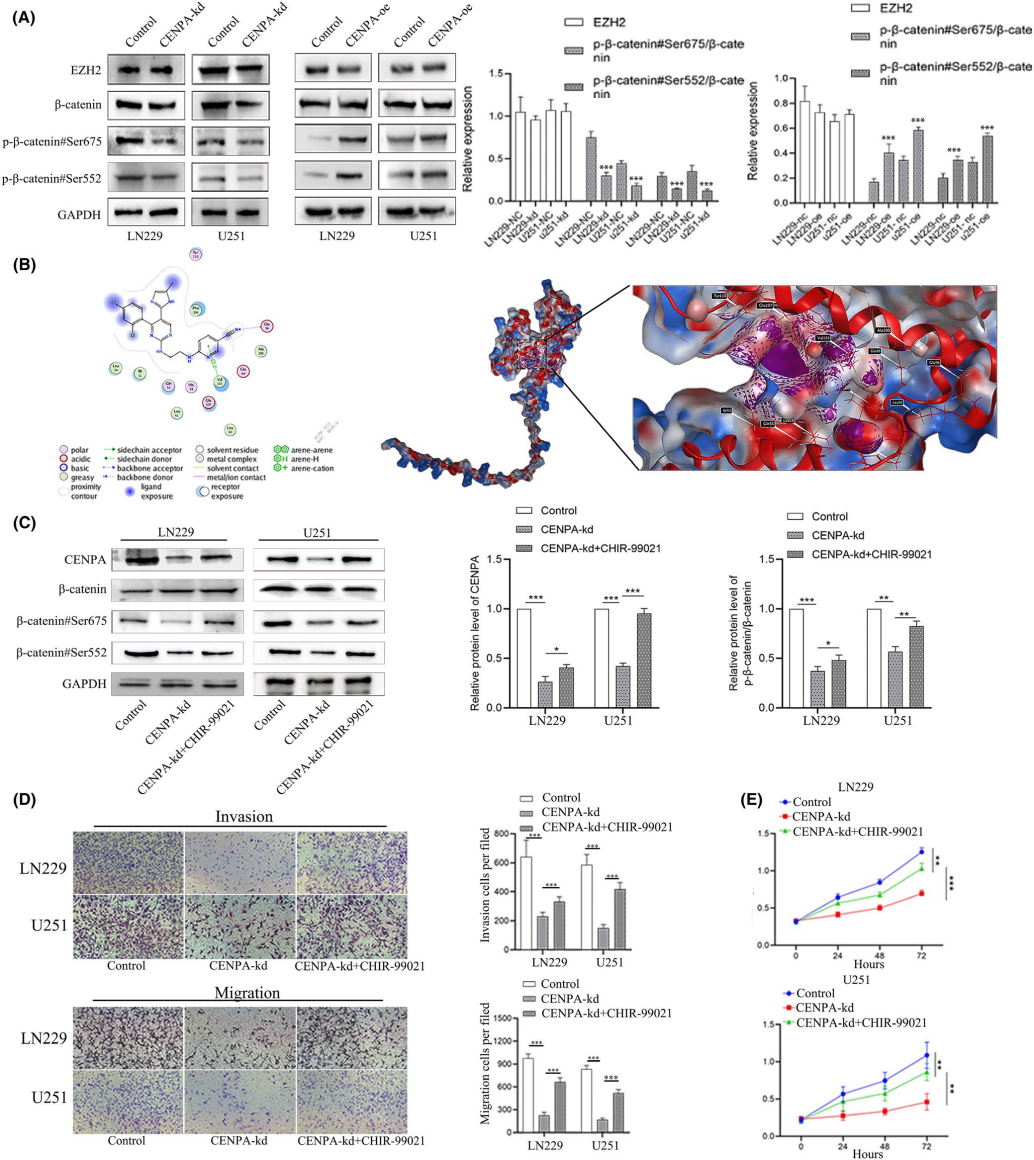
CENPA‐influenced glioma through the wnt pathway. (A) CENPA and wnt pathway through western blot. (B) The combination between CENPA and CHER‐99021 was simulated using MOE software. (C) CHIR‐99021, CENPA and β‐catenin were detected by western blot. (D) Transwell assay was to evaluate the function of CHIR‐99021 for CENPA‐kd cell lines. (E) CCK8 detected CHIR‐99021's function.

EZH2's influence on the Wnt signalling pathway has been widely reported. We hypothesized that CENPA, closely associated with EZH2, might also affect the Wnt signalling pathway. After knocking down CENPA, we found that the total protein level of β‐catenin was not significantly reduced. However, using antibodies against β‐catenin phosphorylation sites at serine 675 and 552, we observed a significant decrease in protein levels at these sites, suggesting that CENPA may promote cancer by affecting the phosphorylation of β‐catenin protein (Figure 9A). Conversely, when CENPA was overexpressed, the expression of phosphorylation sites mentioned above was upregulated, confirming the effect of CENPA on the Wnt pathway (*p* < 0.001, Figure 9A). As a targeted drug for the Wnt pathway, CHIR‐99021 has been widely reported as a Wnt pathway activator. Studies have shown that CHIR‐99021 can penetrate the blood–brain barrier, and data from the MCE drug website also indicate high blood drug concentrations, making it an appropriate choice. After molecular docking of CHIR‐99021 and CENPA using MOE software, the molecular distance between CHIR‐99021 and CENPA was found to be 4.37, with a binding energy of −0.6 kcal/mol and an affinity of −6.037, indicating a relatively tight binding (Figure 9B).

Following administration of CHIR‐99021, there was no significant change in the total protein level of β‐catenin, but phosphorylated protein levels were upregulated. Meanwhile, there was a certain degree of upregulation of CENPA (*p* < 0.05, Figure 9C). Additionally, in both LN229 and U251 cell lines with CENPA knocked down, the addition of CHIR‐99021 reversed the migration, invasion and proliferation abilities of the cells (*p* < 0.001, Figure 9D). Furthermore, after treatment with CHIR‐99021, we found that it partially reversed the effects of CENPA knockdown on cells, improving the reduced tumour proliferation caused by CENPA knockdown (*p* < 0.05, Figure 9E). CENPA not only influenced the Wnt pathway but also had significant effects on other signalling pathways, including the MAPK signalling pathway, cAMP signalling pathway, GABAergic synapse, ECM‐receptor interaction, DNA replication and Hippo signalling pathway (Figure S9), and pathway‐pathway networks could better illustrate the relationships between them (Figure S10). These pathways played critical roles in tumour growth, and we conducted further in‐depth investigations into the key genes involved in these pathways in subsequent studies.

### 3.7 CENPA inhibited the growth of glioma cells in vivio

**FIGURE 10:**
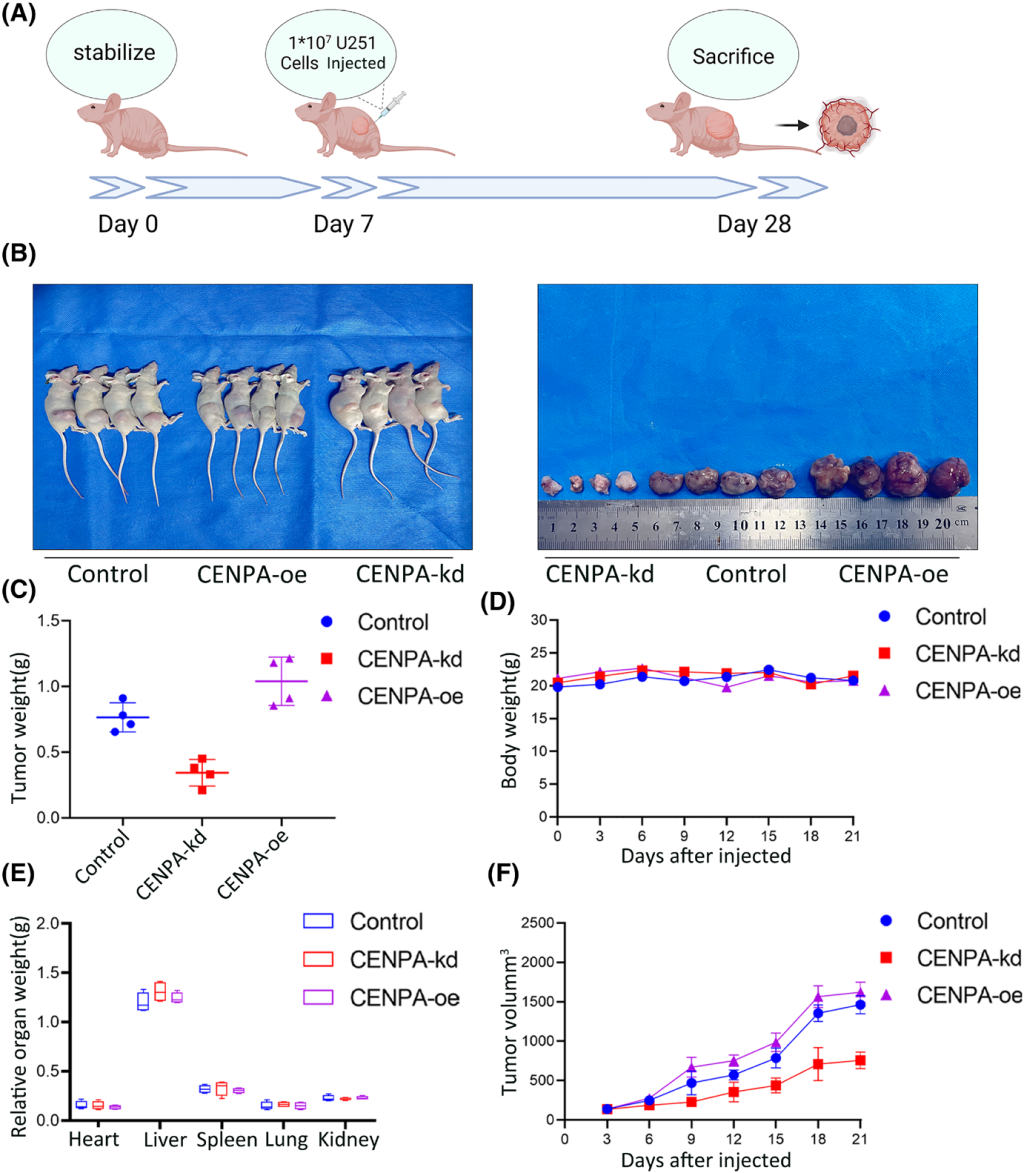
Knockdown and overexpression of CENPA in Balb/c nude mice. (A) Schematic representation of xenograft tumour experiment. (B) Images of xenograft tumours. (C) Tumour volume changes among different groups. (D) Tumour weight at the end of the experiment. (E) Body weight changes of nude mice in different groups. (F) Relative organ weights at the end of the experiment. *p < 0.05, **p < 0.01, ***p < 0.001 compared to the control group.

Subcutaneous tumour experiments were conducted on U251 cells after transfection with lentiviruses for CENPA knockdown and overexpression (Figure 10A). We observed the tumorigenic effects of knocking down CENPA, overexpressing CENPA and control U251 cells. During the first week of the experiment, the differences in tumour volume among the three groups were minor. However, as time progressed, at 2 and 3 weeks, the tumour volume growth rate was slower in the CENPA knockdown group. Still, there were no significant changes in the body weight of nude mice, and limb activity was not markedly affected. The control group and overexpression group showed faster tumour volume growth in nude mice, especially the overexpression group (*p* < 0.001, Figure 10B,C). However, the relative weights, appearance or morphology of organs such as the heart, liver, kidney, lung and spleen showed no significant changes in any of the three experimental groups, perhaps conducting intracranial tumour experiments would be more helpful in observing the results (Figure 10D–F).

## 4 DISCUSSION

In this study, we used 14 malignant features related to glioma to perform unsupervised clustering analysis of glioma samples.^15^ Based on cancer functional states described above, we divided glioma into three subtypes, each with different clinical characteristics, prognosis, immune infiltration, treatment response and somatic mutations. The C subtype exhibited a high level of cancer functional states, with patients showing poor clinical prognosis, high immune cell infiltration, high CNV and tumour mutation burden. CENPA among the A, B and C subtypes was used to construct a risk model feature, improving the performance of prognosis prediction with machine learning. Our study enhanced the understanding of cancer functional states and its diversity, while also broadening the knowledge of molecular subtypes of glioma. The heterogeneity of cancer functional states within tumour regions might affect immune cells around the tumour and might lead to the failure of immunotherapy. Studies suggested that promoting tumour‐associated macrophages could transform into tumour‐supporting phenotypes, mainly due to the accumulation of palisading giant cells induced by hypoxia in glioma, making glioma prone to recurrence.^16^ Here, we conducted unsupervised learning and immune infiltration analysis to reveal unique immune states in CFS subtypes. Our research results indicated that CFS subtypes had different immune features, especially increased immune infiltration in the high C subtype. Additionally, the heterogeneity of CFS in glioma might affect clinical outcomes.^17^ We found that patients with C subtype had the worst prognosis, which might be related to the enrichment of malignant features such as high grade, GBM, wild‐type IDH1, non‐coding 1p19q and old age in the C subtype. Furthermore, the high CNV and tumour mutation burden in the C subtype were also reasons for the poorer clinical prognosis. We speculated that the reason for the high CNV was DNA fragment deletion and amplification, local chromosomal rearrangement, extrachromosomal DNA and micronucleus formation.^18^ These results indicated that cancer functional states subtypes were closely related to clinical characteristics. Combining nearly 20 years of research on gene mutations and cancer functional states, it could be concluded that oncogenic drivers were the main cause of cancer functional states progression. IDH1^19^ mutations activated the mTOR signalling pathway, promoting the proliferation and invasion of glioma cells. Glioma cells could be sensitive to hypoxia, radiation and temozolomide by downregulating p53‐induced glycolysis and apoptosis regulators.^20^, ^21^ High‐grade glioma without EGFR mutations showed increased vascular density^22^; however, due to reduced BMX/SOX9^23^ activity and insufficient coverage of perivascular cells, the blood–brain barrier was highly disrupted, leading to hypoxia, necrosis and ischemia. All of these oncogenic drivers contributed to the heterogeneity of cancer functional states within glioma.

Using cross‐genes between cancer functional states subtypes, we constructed a prediction model with 101 prediction models based on machine learning, screening out five oncogenes and further experiments confirmed centromere protein A (CENPA). The study found that the expression level of CENPA was generally upregulated in various human cancer types, including but not limited to breast cancer,^12^ colorectal cancer,^24^ liver cancer,^10^ lung adenocarcinoma,^13^ ovarian cancer,^11^ and prostate cancer.^25^ Analysis of multiple studies revealed that compared to healthy tissues, the expression level of CENPA was significantly upregulated in 20 solid tumours.^7^ For instance, in oestrogen receptor‐positive breast cancer patients who did not receive chemotherapy, CENPA could serve as an independent prognostic marker, and it was positively correlated with the Ki‐67 proliferation marker, which was also verified in the study of luminal A‐type breast cancer.^26^ CENPA could also activate downstream CCND1 and NRP2 by binding with the transcription factor YY1, thereby affecting the proliferation and invasion of hepatocellular carcinoma.^10^ Our experiments first verified the expression of CENPA in TCGA and CGGA databases, tissue samples and cell lines, confirming its upregulation in glioma tissues according to WHO grading. We found that CENPA affected the proliferation of glioma. Studies have suggested that during cell mitosis, CENPA can promote cells from the G1 phase to the S phase by affecting the expression of CCND1 and CDK4/6 proteins.^10^ In addition to these proteins, we also observed an increase in the level of p21 protein when CENPA was knocked down. Besides being closely associated with p53‐MDM2, p21 mainly inhibits the cell cycle process by inhibiting the activity of CDK.^27^ Furthermore, we confirmed through the TCGA, CGGA and Rembrandt databases that the overexpression of CENPA affected the EMT phenotype. Transcription factors such as Snail, Slug and Twist were significantly associated with CENPA, which might enhance the invasive migration ability of glioma, adversely affecting the prognosis. To further validate this, we performed scratch and Transwell assays and found that the migration and invasion abilities of LN229 and U251 cells were significantly enhanced after overexpression of CENPA, and the specific mechanism needed further study.

To explore the mechanism of CENPA's influence on cancer, we searched for EZH2 as an upstream factor of CENPA. Biologically, EZH2, besides being a histone methyltransferase, can also activate or inhibit the transcription of some genes.^28^, ^29^ For example, EZH2 can inhibit the expression of CDKN1A (encoding p21) and CDKN2A (encoding p16), regulate the expression of some transcription factors such as FOXC1,^30^ TWIST1,^31^ and SNAIL,^32^ and the expression of BLC2 apoptosis family proteins,^33^ thus playing an important role in tumorigenesis and development. Considering that EZH2 might regulate CENPA, using AlphaFold^34^ and COIP experiments, we found that the dissociation constant between EZH2 and CENPA was high, indicating interaction between them. Furthermore, considering EZH2's influence on the Wnt signalling pathway,^35^ experiments confirmed that CENPA also affected the key protein β‐catenin in the Wnt pathway, and had a significant positive feedback effect on the phosphorylation sites of β‐catenin, Ser675 and Ser552. These two phosphorylation sites promote the accumulation of β‐catenin in the cell nucleus.^36^, ^37^ We considered that when the binding of CENPA to DNA decreased, the stability of DNA bound to β‐catenin might decrease, leading to transcriptional regulation inhibition in the nucleus, and ineffective β‐catenin being ubiquitinated and degraded. After knocking down CENPA, the cell cycle was inhibited. For example, the regulation of CENPA could affect CCND1 and CDK4/6, interfering with cell cycle, and also affect the activity of the Wnt signalling pathway, ultimately affecting the proliferation and growth of tumour cells. We chose CHIR‐99021 as a Wnt pathway activator to further verify the relationship between CENPA and the Wnt pathway because CHIR‐99021 has high drug solubility and is a small molecule widely used in the study of the Wnt/β‐catenin signalling pathway.^38^ In addition to affecting β‐catenin, MOE protein molecular docking software suggested that CHIR‐99021 could bind to CENPA, which was confirmed in our experiments. This indicates that CHIR‐99021 can affect CENPA to further affect the Wnt pathway. Our research found that CENPA also influenced signalling pathways and related key genes involved in nuclear division, DNA replication, the Hippo signalling pathway, the MAPK signalling pathway and others. The analysis suggested that CENPA significantly affected other cancer‐related signalling pathways except for the Wnt signalling pathway. This implied that research in the future could explore multiple directions to further elucidate the relationship between CENPA and glioma. Furthermore, by searching for pathway inhibitors and activators, drugs targeting CENPA might be found. Currently, studies often involve the combined use of multiple drugs,^39^ which will help solve problems such as drug resistance in glioma cells.

This experiment integrates multiple fields, including cancer functional states, risk model construction, machine learning and pharmacology, presenting a diverse and comprehensive research method. Cancer functional states as a gene screening feature is an innovative approach that can more comprehensively understand the role of genes in tumours and provide more information for subsequent machine learning models. Using risk model construction and machine learning methods to screen target genes is an efficient way. The combination of bioinformatics and pharmacology can help identify upstream and downstream genes related to target genes and potential targeted drugs.^40^

## 5 CONCLUSION

Machine learning based on CFS is beneficial for the selection of key genes and disease assessment. In glioma, CENPA is positively correlated with WHO grading at both the gene and protein levels, and high CENPA affects patients' poor prognosis. Regulating CENPA can affect functions of glioma, and these phenomena may act through the EZH2/CENPA/β‐catenin signalling axis. CENPA knockdown can be reversed by the drug CHIR‐99021. CENPA may become one of the therapeutic targets in glioma.

## AUTHOR CONTRIBUTIONS

**Yuanguo Ling:** Conceptualization (equal); data curation (equal); formal analysis (equal); resources (equal); software (equal); supervision (equal). **Wei Teng:** Conceptualization (equal); data curation (equal); formal analysis (equal); investigation (equal); methodology (equal). **Niya Long:** Conceptualization (equal); data curation (equal); methodology (equal); validation (equal). **Wenjin Qiu:** Conceptualization (equal); data curation (equal); resources (equal); software (equal). **Ruting Wei:** Conceptualization (equal); data curation (equal); resources (equal); software (equal). **Yunan Hou:** Conceptualization (equal); data curation (equal). **Lishi Jiang:** Methodology (equal); project administration (equal); supervision (equal); validation (equal). **Jian Liu:** Funding acquisition (equal); investigation (equal). **Xingwang Zhou:** Data curation (equal); formal analysis (equal). **Liangzhao Chu:** Conceptualization (equal); data curation (equal); funding acquisition (equal); software (equal); supervision (equal).

## FUNDING INFORMATION

This study was funded by Guizhou Province Science and Technology Plan Project (Project) Qiankehe Foundation‐ZK [2023] General 362 to Liangzhao Chu; Science and Technology Fund project of Guizhou Provincial Health Commission (gzwkj‐2022‐09) to Liangzhao Chu; National Natural Science Foundation Cultivation Project of Guizhou Medical University (21NSFCP14) to Liangzhao Chu; National Natural Science Foundation of China (Number: 82360493) to Xingwang Zhou; Guizhou Province Science and Technology Plan Project (Project) Qiankehe Foundation‐ZK (2023) General 360 to Niya Long; National Natural Science Foundation Cultivation Project of Guizhou Medical University (gyfynsfc‐2022‐25) to Niya Long; Science and Technology Fund project of Guizhou Provincial Health Commission (gzwkj‐2023‐035) to Niya Long; The PhD scientific research launch fund project of the Affiliated Hospital of Guizhou Medical University (gyfybsky‐2022‐02) to Niya Long.

## CONFLICT OF INTEREST STATEMENT

The authors declare no conflict of interest.

## CONSENT FOR PUBLICATION

Written informed consent was obtained from the patient for publication of this case report and any accompanying images. A copy of the written consent is available for review by the Editor‐in‐Chief of this journal.

## Supporting information


Figure S1.

Figure S2.

Figure S3.

Figure S4.

Figure S5.

Figure S6.

Figure S7.

Figure S8.

Figure S9.

Figure S10.

## Data Availability

The authors confirm that the data supporting the findings and conclusions of this study are available in the article.
