# Supplementary figures and images for: Exploration of the biological mechanisms of CENPA as an oncogene in glioma: Screening based on cancer functional status
Source: J Cell Mol Med. 2024 Dec 2;28(23):e70181. doi: 10.1111/jcmm.70181 (PMC11610157; doi:10.1111/jcmm.70181)

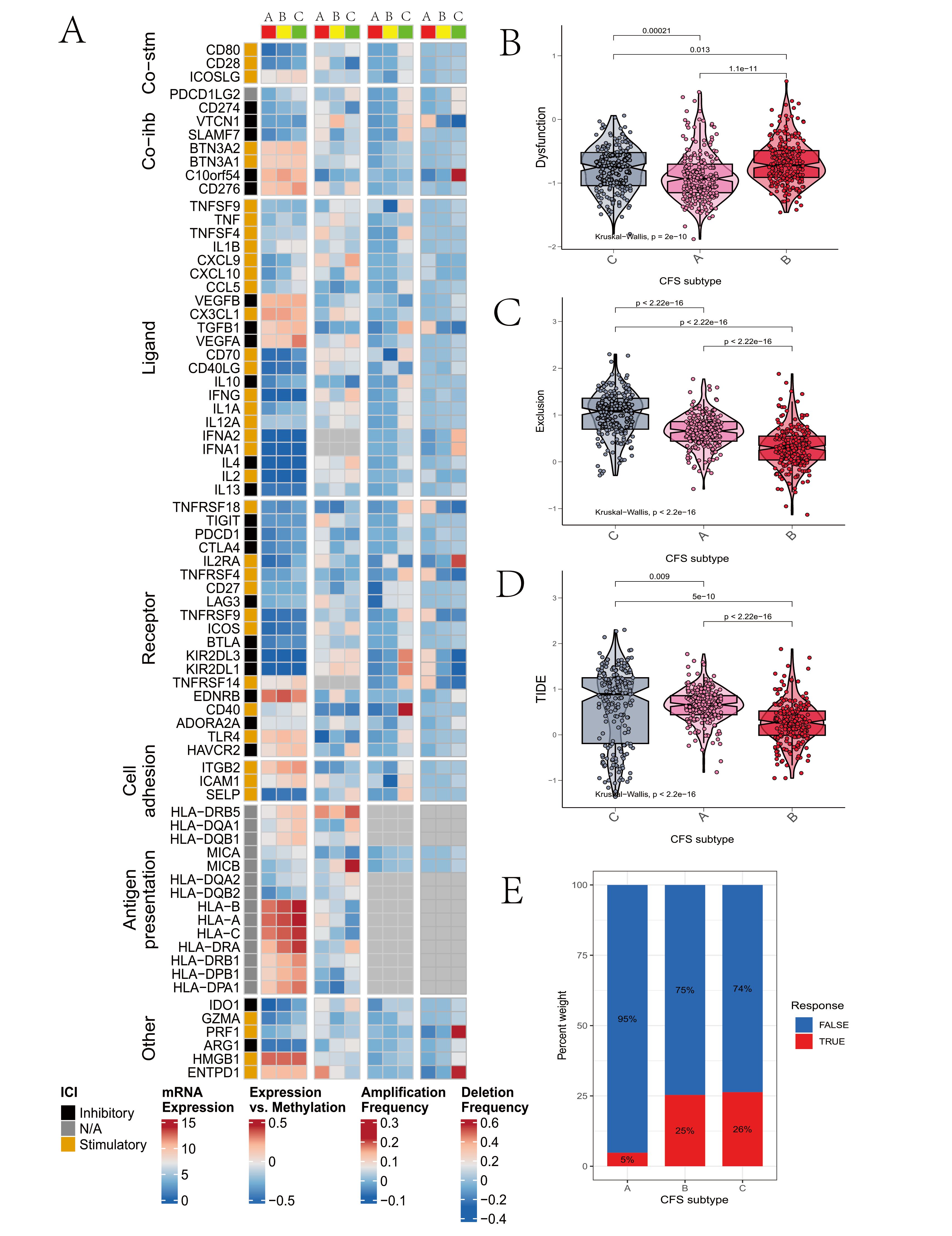

Supplement: Supplementary file 1 — Figure S1. [file JCMM-28-e70181-s007.jpg]

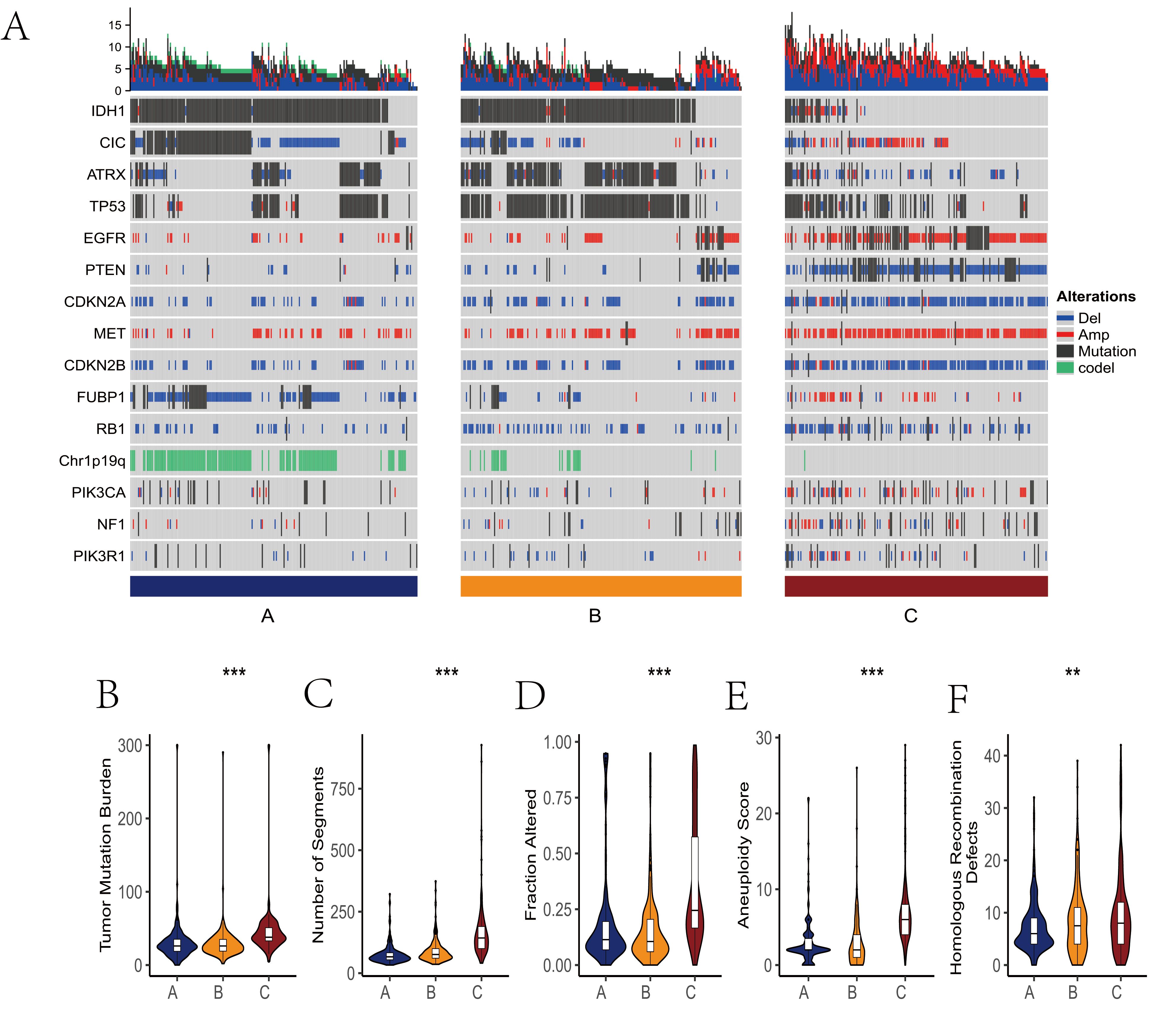

Supplement: Supplementary file 2 — Figure S2. [file JCMM-28-e70181-s008.jpg]

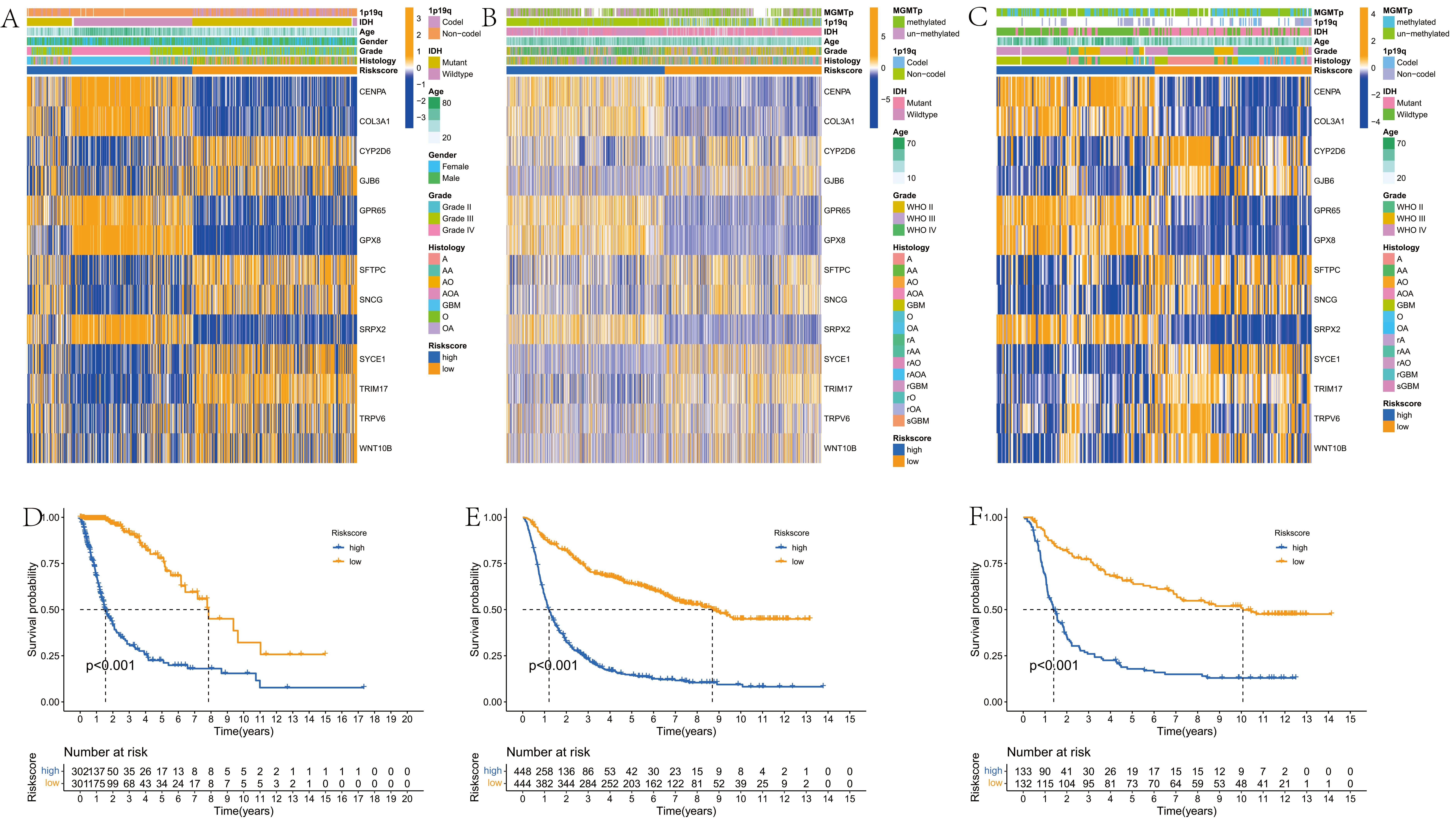

Supplement: Supplementary file 3 — Figure S3. [file JCMM-28-e70181-s002.jpg]

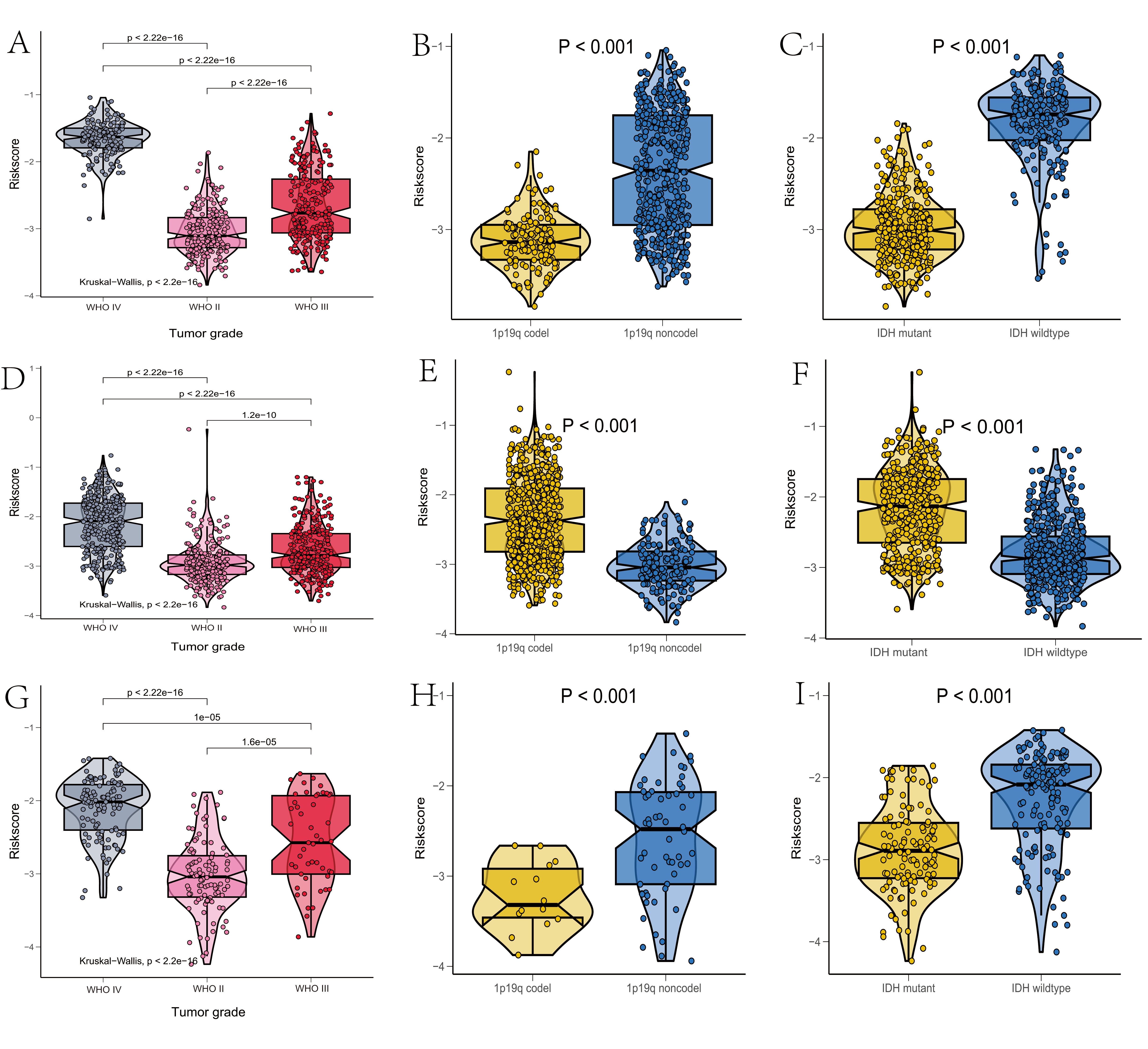

Supplement: Supplementary file 4 — Figure S4. [file JCMM-28-e70181-s003.jpg]

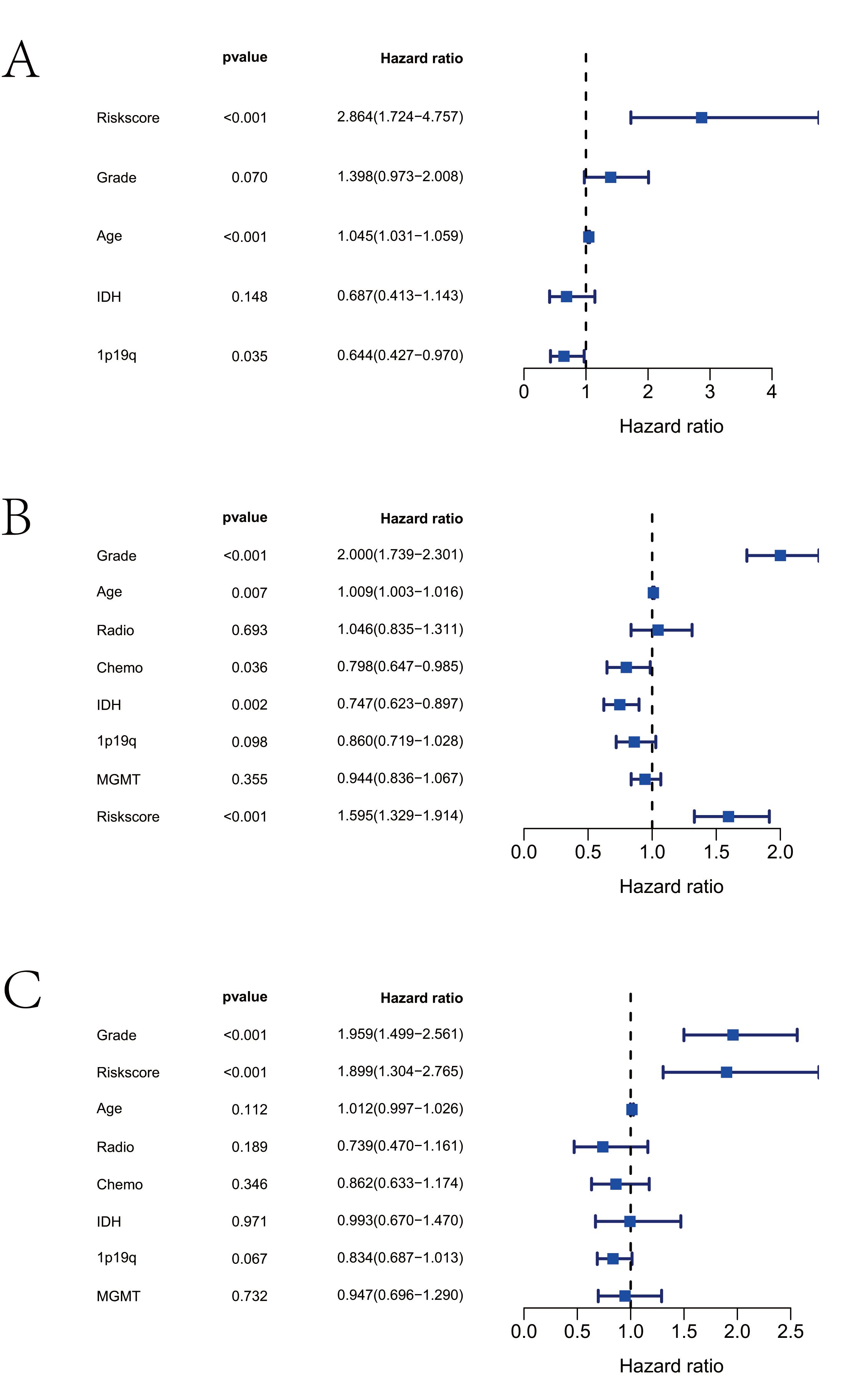

Supplement: Supplementary file 5 — Figure S5. [file JCMM-28-e70181-s001.jpg]

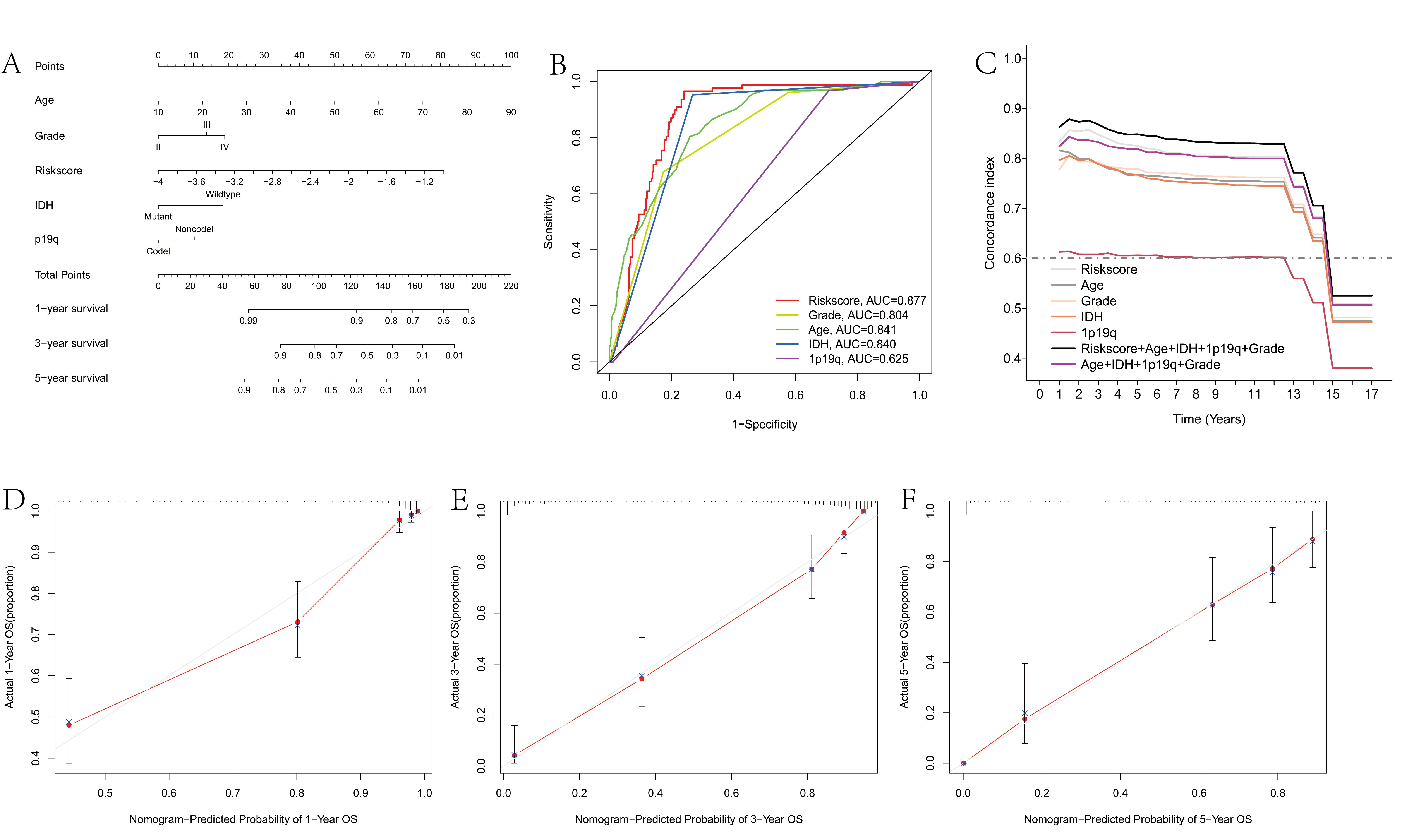

Supplement: Supplementary file 6 — Figure S6. [file JCMM-28-e70181-s009.jpg]

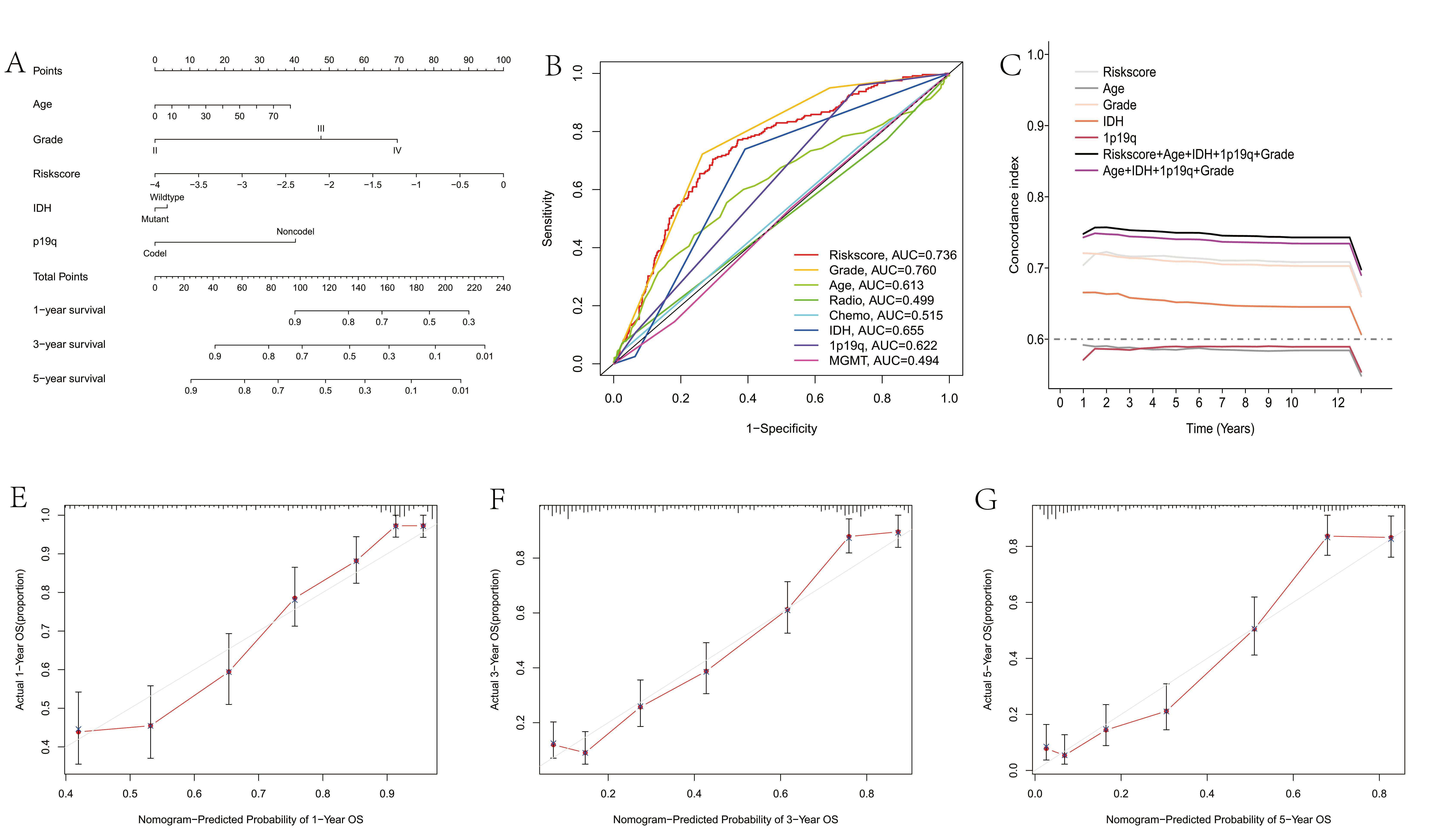

Supplement: Supplementary file 7 — Figure S7. [file JCMM-28-e70181-s010.jpg]

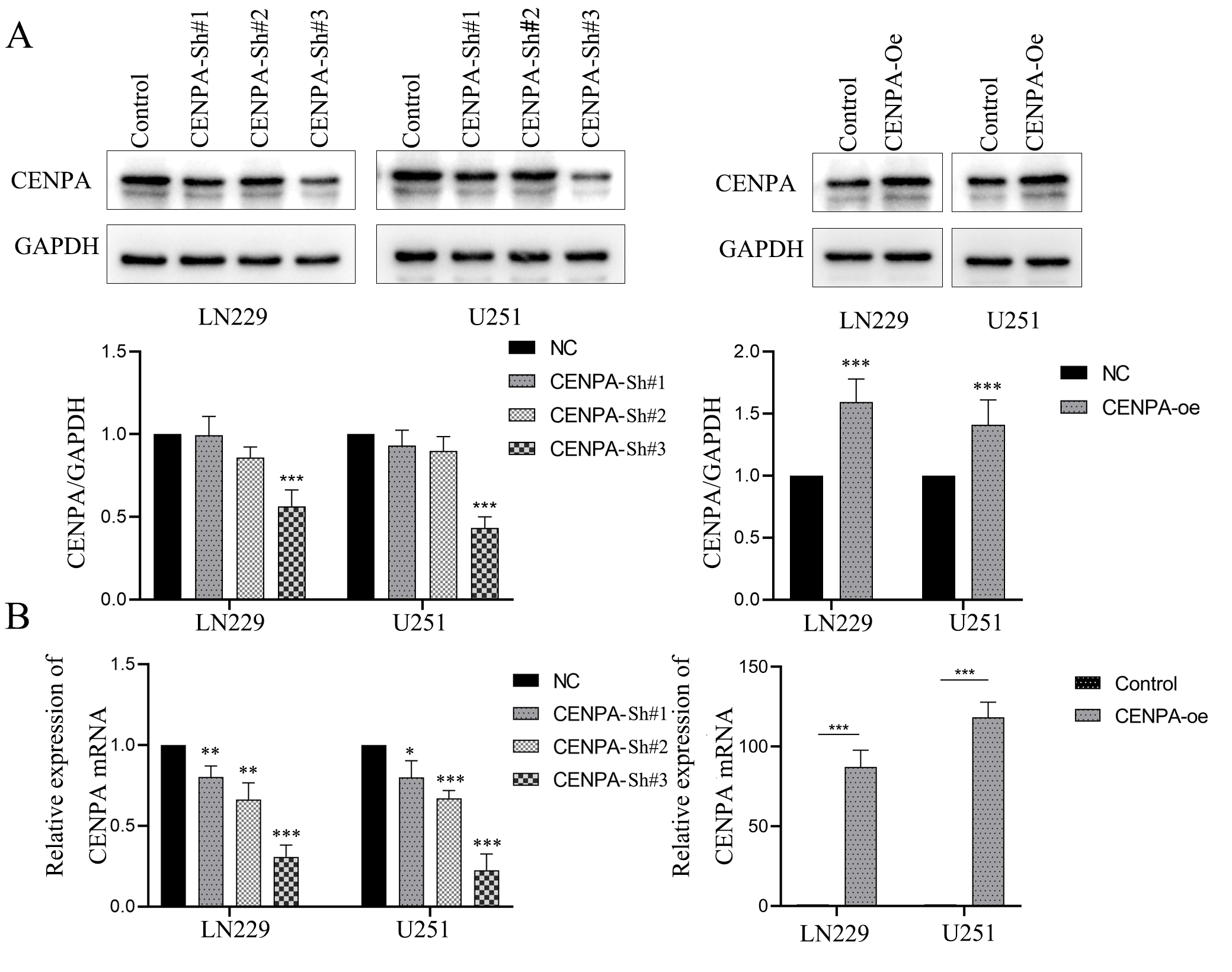

Supplement: Supplementary file 8 — Figure S8. [file JCMM-28-e70181-s004.png]

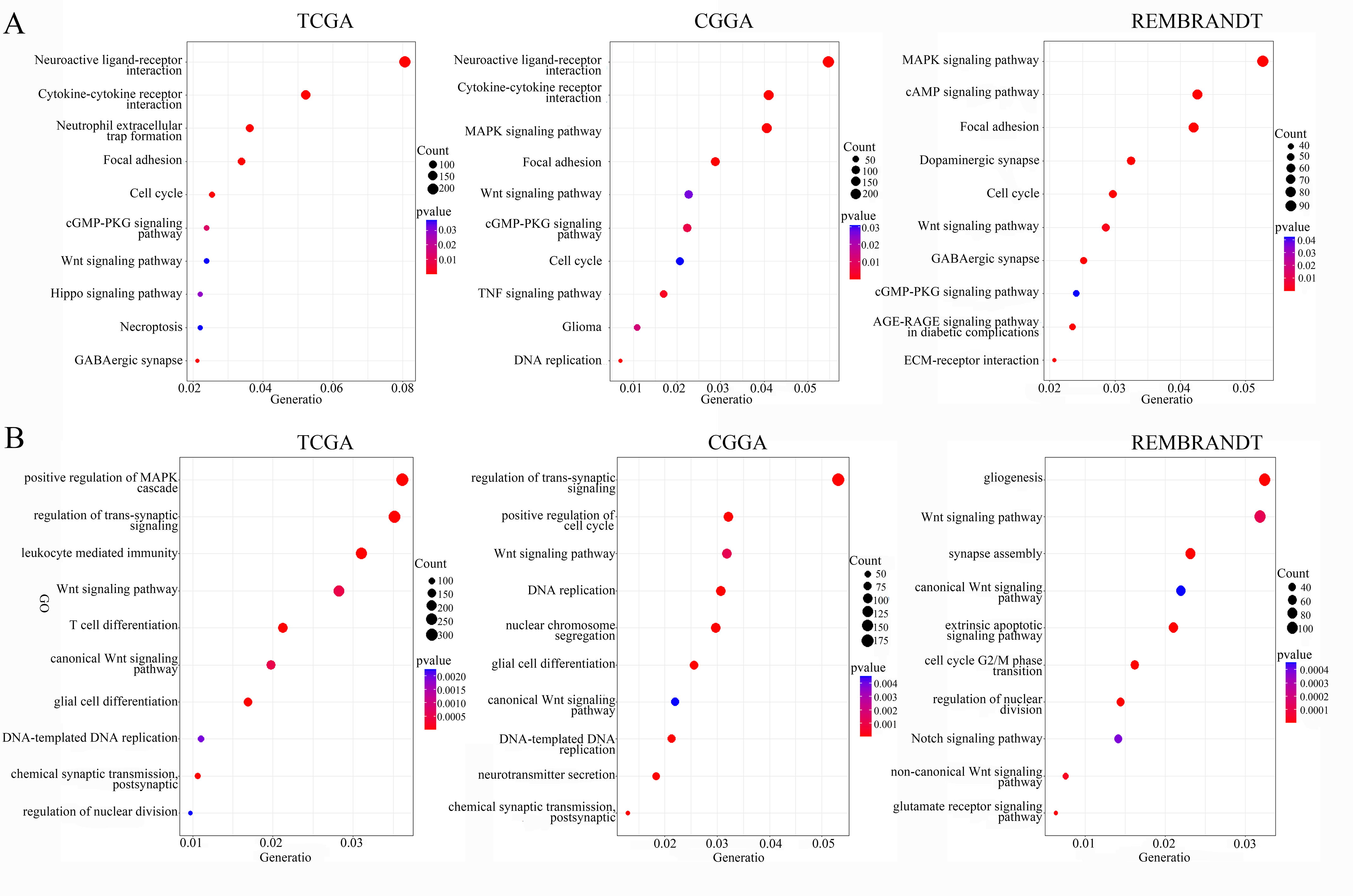

Supplement: Supplementary file 9 — Figure S9. [file JCMM-28-e70181-s005.png]

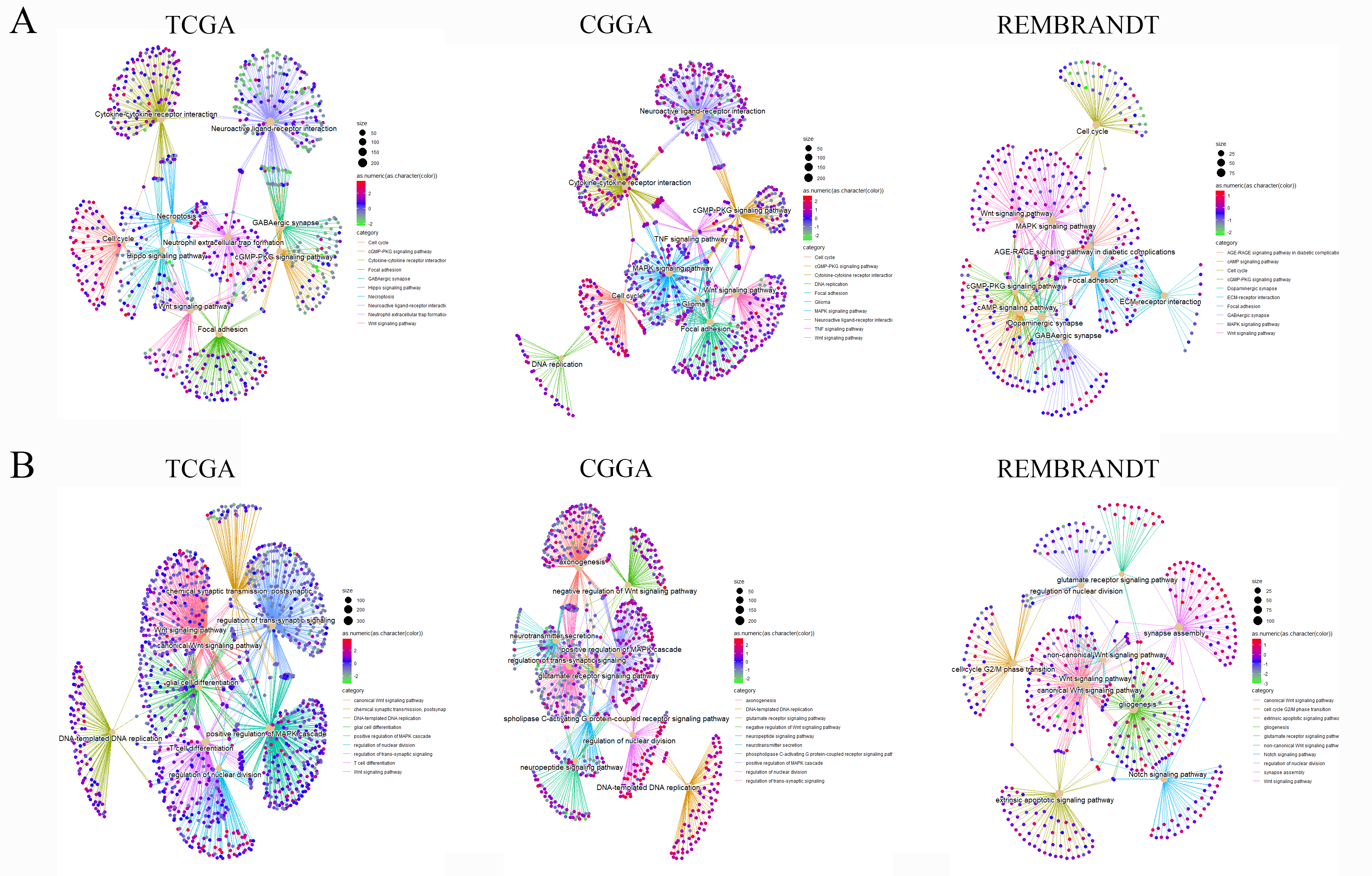

Supplement: Supplementary file 10 — Figure S10. [file JCMM-28-e70181-s006.png]
